# A targeted analysis reveals relevant shifts in the methylation and transcription of genes responsible for bile acid homeostasis and drug metabolism in non-alcoholic fatty liver disease

**DOI:** 10.1186/s12864-016-2814-z

**Published:** 2016-06-14

**Authors:** Helgi B. Schiöth, Adrian Boström, Susan K. Murphy, Wiebke Erhart, Jochen Hampe, Cynthia Moylan, Jessica Mwinyi

**Affiliations:** Department of Neuroscience, Division of Functional Pharmacology, Uppsala University, Uppsala, Sweden; Department of Obstetrics and Gynecology, Duke University Medical Center, Durham, NC USA; Department of Internal Medicine I, University Hospital Schleswig-Holstein, Kiel, Germany; Medical Department I, University Hospital Dresden, Dresden, Germany; Department of Medicine, Duke University Medical Center, Durham, NC USA; Department of Medicine, Durham Veterans Affairs Medical Center, Durham, NC USA

**Keywords:** Liver, NAFLD, Methylation, Bile acid homeostasis, Drug metabolizing enzymes, Drug transporters, Correlation of methylation and transcriptional expression

## Abstract

**Background:**

Non-alcoholic fatty liver disease (NAFLD) is associated with a high risk for liver cirrhosis and cancer. Recent studies demonstrate that NAFLD significantly impacts on the genome wide methylation and expression reporting top hit genes to be associated with e.g. diabetes mellitus. In a targeted analysis we specifically investigate to what extent NAFLD is associated with methylation and transcriptional changes in gene networks responsible for drug metabolism (DM) and bile acid (BA) homeostasis, which may trigger liver and system toxic events.

**Methods:**

We performed a systematic analysis of 73 genes responsible for BA homeostasis and DM based on liver derived methylation and expression data from three cohort studies including 103 NAFLD and 75 non-NAFLD patients. Using multiple linear regression models, we detected methylation differences in proximity to the transcriptional start site of these genes in two NAFLD cohorts and correlated the methylation of significantly changed CpG sites to transcriptional expression in a third cohort using robust multiple linear regression approaches.

**Results:**

We detected 64 genes involved in BA homeostasis and DM to be significantly differentially methylated. In 26 of these genes, methylation significantly correlated with RNA expression, detecting i.e. genes such as *CYP27A1*, *OSTɑ*, and *SLC27A5* (BA homeostasis), and *SLCO2B1, SLC47A1,* and several *UGT* and *CYP* genes (DM) to be NAFLD dependently modulated.

**Conclusions:**

NAFLD is associated with significant shifts in the methylation of key genes responsible for BA and DM that are associated with transcriptional modulations. These findings have implications for BA composition, BA regulated metabolic pathways and for drug safety and efficacy.

**Electronic supplementary material:**

The online version of this article (doi:10.1186/s12864-016-2814-z) contains supplementary material, which is available to authorized users.

## Background

Non-alcoholic fatty liver disease (NAFLD) is the most common chronic liver disorder in industrialized countries with a 20–40 % prevalence worldwide [1]. While the milder form of NAFLD, characterized by simple steatosis (SS) is generally benign, the progressive form, nonalcoholic steatohepatitis (NASH), distinguished by necroinflammation and fibrosis, is linked to an increased risk for the development of cirrhosis, hepatic failure and hepatocellular carcinoma [2]. The exact pathogenesis of NAFLD, especially the progression from SS to NASH remains unclear but is hypothesized to be triggered by proinflammatory cytokines and intrahepatic accumulation of saturated fatty acids and cholesterol [3–7].

Epigenetic mechanisms, including changes in methylation patterns or histone modifications, are able to efficiently change the expression of genes by modulating the accessibility of gene regulatory regions by transcription factors [8]. Studies have repeatedly demonstrated that changes in methylation patterns are able to modify the risk for metabolic diseases, such as e.g. obesity [9], or diabetes mellitus [10, 11]. Methylation patterns underlie dynamic changes, thus, being suggested to influence disease course and disease severity [12].

Three genome wide studies recently elucidated major NAFLD and NASH associated shifts in the methylome and transcriptome and gave first important insights into the genome wide impact of NALFD on these gene regulatory mechanisms [8, 13, 14]. Ahrens et al. NAFLD investigated methylation and associated transcriptional changes in liver tissue after bariatric surgery and detected as top hits methylation changes in genes involved in intermediate metabolism and insulin dependent signaling pathways. Murphy et al. and Moylan et al. demonstrated in two studies that especially progression of NAFLD leads to the induction of genome-wide occurring changes in methylation and associated changes in the transcriptome, which enables the distinction between mild fibrotic and advanced fibrotic NAFLD disease stages. Comparing patient with mild and advanced fibrosis, the authors showed that key genes associated with diabetes mellitus, cardiovascular disease and cancer are significantly modulated in methylation and expression during NAFLD progress.

No study has to date addressed the question, to what extent NAFLD is specifically related to epigenetic and transcriptional shifts in gene networks responsible for bile acid (BA) homeostasis and drug metabolism. Disease associated changes in the expression of those genes may have relevant consequences for lipid homeostasis, liver function and drug therapeutic efficacy. Synthesized from cholesterol, bile acids (BAs) are the main source of cholesterol catabolism in humans and are responsible for the intestinal uptake of fat soluble compounds. They are main ligands to the nuclear receptor FXR that has been shown to improve the lipid and glucose profile and has been repeatedly discussed as a putative drug target for the treatment of NAFLD [15]. Thus, BA are important regulators of cholesterol and lipid homeostasis. Importantly, BA homeostasis is tightly regulated by FXR. FXR is known to control every step in BA synthesis, transport and metabolism (reviewed in [16]). This comprises the conjugation of BA to taurine and glycine and the enterohepatic circulation via several hepatic and intestinal FXR regulated influx and efflux transporters, such as bile salt export pump (BSEP), Apical Sodium-dependent Bile Acid Transporter (ASBT), organic solute transporter (OSTα/β), the sodium (Na)-Taurocholate Cotransporter Protein (NTCP) and the organic anion transport peptide 1B3 (OATP1B3). FXR-regulated enzymes, including cytochrome P450 (CYP) 7A1, CYP8B1 and CYP27A1, CYP3A4, CYP3A11, sulphotransferase 2A1 (SULT2A1) and UDP-glucuronosyltransferase 2B4 (UGT2B4/UGT2B11) participate in the synthesis and metabolism of BAs. Imbalances in the composition of the BA pool have the potential to induce and to potentiate hepatotoxicity through pro-inflammatory mechanisms, membrane damage and cytotoxic reactions and may have consequences for lipid homeostasis [17, 18]. Lake et al. recently demonstrated that the BA composition in NASH patients is shifted towards taurine conjugated BA derivatives, which was hypothesized to be caused by observed expression changes in several BA transforming proteins [19]. BA homeostasis and drug metabolism (DM) and disposition are closely linked to each other. BAs are not only targeting FXR but also other nuclear receptors, such as e.g. PXR and CAR, thereby impacting the transcriptional regulation of many proteins involved in drug transport and transformation. Furthermore, several enzymes and transporters involved in BA homeostasis transport transform therapeutics as well [20]. This includes amongst others the hepatic uptake transporters OATP1B1 and OATP1B3, the efflux transporters MRP2 and MDR1 and metabolizing enzymes, such as CYP3A4 or UGT2B11.

Using three independent NAFLD cohorts, we specifically address for the first time the question to what extent NALFD shows systematic shifts in the epigenetic profile of genes regulating BA homeostasis and drug metabolism and transport with relevance for the transcriptional expression in both gene networks. In this context we detect and define epigenetic surrogate markers in proximity of the transcriptional start site in two gene networks responsible for BA and DM that are sensitive to NAFLD and NAFLD progression and of functional relevance for transcription. The study provides a novel insight into pathogenetic mechanisms underlying a changed BA composition in NAFLD and the risk for changes in drug metabolism and disposition in NAFLD, which both may lead to a potentiation of harmful effects on the liver.

## Results

### NAFLD is associated with significant methylation changes in genes involved in BA homeostasis and drug metabolism

**Fig. 1:**
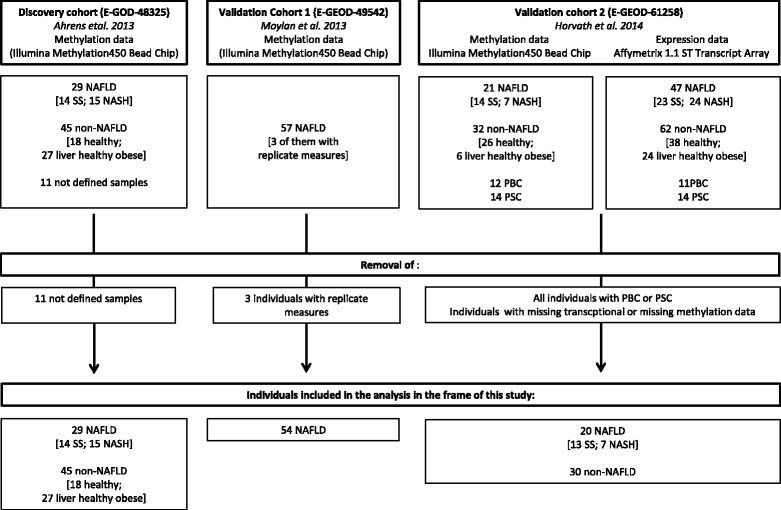
Flowchart showing the initial subject composition of all three cohorts included into the study as well as number of and reasons for sample dropouts

Based on genome wide methylation data measured in three independent NAFLD cohorts we specifically investigated and compared the methylation profiles of genes associated with BA transport and metabolism (gene cluster 1, 43 genes, Table 2) and drug transport and metabolism (gene cluster 2, 40 genes, Table 2) between 29 NAFLD patients (i.e., SS (*n* = 14) and NASH (*n* = 15)) and 45 non-NAFLD control patients (discovery group, [8], (E-GEOD-48325), Table 1, Fig. 1) using multiple linear regression analyses. For these analyses we specifically included all CpG sites located within the area 1500 bp upstream and downstream from the transcriptional start site (TSS1500 interval) of each gene were taken into consideration. Thirty three genes in cluster 1 and 31 genes in cluster 2 were significantly differentially methylated between NAFLD and controls (Additional files 1: Table S1 and Additional file 2: Table S2). Using multiple linear regression analyses the detected differential methylation of these genes was confirmed in a second independent cohort investigating the methylation state of these genes in dependency of fibrosis severity in 54 patients suffering from SS or NASH (validation cohort, [13], E-GEOD-49542, Table 1, Fig. 1). Additional files 1: Table S1 and Additional file 2: Table S2 show all significantly differentially methylated genes and associated CpG sites that were confirmed in the validation cohort. Tables 3 and 4 show an excerpt of the supplemental tables, condensing the list to those genes and associated CpG sites that were later on shown to correlate with gene expression. As demonstrated in (Additional files 3: Table S3 and Additional file 4: Table S4) methylation changes related to NAFLD range from 3 % to 6 % for the majority of CpG sites. Especially strong methylation shifts at distinct CpG sites between mild and advanced stages of NAFLD in genes showing methylation-transcription associations (Table 5 and 6) were observed for *SLCO2B1*, *EPHX* (7 % each), and *ATP8B1* (13 %, gene cluster 1) and for *UGT1A1*, *GSTP1* (7 %), *UGT1A4* (8 %), *SLC47A1, CYP1A2* (9 % each), *SULT1A1* (10 %), *CYP1A1* (12 %) and *CYP2C19* (15 %, also mentioned in [8]) (gene cluster 2). Genes harboring CpG sites that were significantly modulated in their methylation profile encode for proteins that belong to all different functional categories represented in both gene clusters as distinguished in Table 2. This includes BA synthetizing, modulating, metabolizing and transporting genes, drug metabolizing and transporting genes as well as several important gene regulating nuclear receptors. Methylation changes observed in both gene clusters in our targeted analysis are of considerable extent. Ahrens et al., describes that the hepatic differential methylation of genome-wide top-hit findings before and after bariatric surgery lies between 5 % and 15 % before and after bariatric surgery [8]. Murphy and co-workers highlight especially three genes of interest in their genome-wide analyses (*FGFR2*, *MAT1A* and *CASP1*) that vary between 2 % and 5 % in methylation between mild and advanced NAFLD cases [13].

**Table 1:** Characteristics of individuals included into the study

	Discovery Group (*n* = 74)			Validation Cohort^a^ (*n* = 54)		Expression correlation (EC) cohort		
						Expr. Analysis Cohort (*n* = 50)		
Parameter	Control	SS	NASH	Control	SS & NASH	Control	SS	NASH
*n*	45	14	15	0	54	30	13	7
Men:Women (n (%))	8 (17.8):37 (82.2)	4 (28.6):10 (71.4)	4 (26.7):11 (73.3)	N/A	16 (29.6):38 (70.4)	15 (50.0):15 (50.0)	9 (69.2):4 (30.8)	7 (100.0):0 (0.0)
Age (years, Mean (SD))	49.2 (14.0)	46.5 (13.9)	45.1 (8.8)	N/A	51.2 (10.0)	62.1 (16.2)	41.3 (5.9)	53.9 (9.5)
Body mass index (BMI, Mean (SD))	33.9 (10.6)	48.5 (7.0)	48.5 (12.4)	N/A	35.9 (8.8)	26.6 (6.4)	53.9 (9.5)	52.5 (11.2)
Diabetes mellitus Type 2	36 no;4 nk;5 yes	9 no; 2 nk; 3 yes	10 no; 3 nk; 2 yes	N/A	36 no; 18 yes	Diabetic state nk		
Fibrosis stage (n (%))^b^								
Insignificant	N/A	N/A	N/A	N/A	0 (0.0)	3 (10.0)	10 (76.9)	4 (57.1)
Mild	N/A	N/A	N/A	N/A	32 (59.3)	0 (0.0)	3 (23.1)	2 (28.6)
Advanced	N/A	N/A	N/A	N/A	22 (40.7)	0 (0.0)	0 (0.0)	1 (14.3)
Steatosis Grade^c^								
< 5 %	N/A	N/A	N/A	N/A	4 (7.4)	23 (76.6)	0 (0.0)	0 (0.0)
5–33 %	N/A	N/A	N/A	N/A	23 (42.6)	7 (23.3)	2 (15.4)	0 (0.0)
34–66 %	N/A	N/A	N/A	N/A	16 (29.6)	0 (0.0)	5 (38.5)	1 (14.3)
> 66 %	N/A	N/A	N/A	N/A	11 (20.4)	0 (0.0)	5 (38.5)	5 (71.4)
missing							1	1

*n* absolute number, *SD* standard deviation, *N/A* not applicable, *nk* not known

^a^An exact subdivision NAFLD into SS or NASH was not provided in ArrayExpress

^b^Severity of fibrosis, evaluated as 0, 0.5, 1, 2 or N/A in the EC cohort, was matched to the fibrosis stage in the validation cohort (insignificant (non-fibrotic samples were not available in this cohort), mild, advanced) as following: ‘Mild’ (1), ‘Advanced’ (2), and ‘Insignificant’ (0 or 0.5)

^c^The strength of steatosis, defined as 0, 1, 2, 3 or N/A in the EC cohort was matched to the degree of steatosis in the validation cohort as following: ' < 5 %', 1 = '5–33 %', 2 = '34–66 %' and 3 = ' > 66 %'

**Table 2:** Genes investigated for NAFLD associated changes in methylation

Bile acids (gene cluster 1)				Drug metabolism (gene cluster 2)			
Transport	Synthesis and metabolism	Signaling and gene regulation	Conjugation	Phase I metabolism	Phase II metabolism	Transporters	Regulation of expression
*ABCB1*	*CYP27A1*	*FGF19*	*BAAT*	*CYP1A1*	*UGT1A1*	*SLC22A1*	*HNF1α*
*ABCB11*	*CYP39A1*	*FGFR4*	*SLC27A5 (BACS)*	*CYP1A2*	*UGT1A4*	*SLC22A2*	*NR2A1 (HNF4α)*
*ABCB4*	*CYP3A4*	*NR1H4 (FXR)*		*CYP2A6*	*UGT2B7*	*SLC22A6*	*AhR*
*ABCC2*	*CYP7A1*	*HNF1α*		*CYP2B6*	*SULT1A1*	*SLC22A7*	*NR1I2 (PXR)*
*ABCC3*	*CYP7B1*	*NR2A1 (HNF4α)*		*CYP2C8*	*GSTM1*	*SLC22A8*	*NR1I3 (CAR)*
*ABCC4*	*CYP8B1*	*NR0B2*		*CYP2C9*	*GSTP1*	*SLC47A1*	*NR3C1 (GR)*
*ABCG1*	*EPHX*	*NR1I2 (PXR)*		*CYP2C19*	*GSTT1*	*SLCO1A2*	*PPARγ*
*ABCG5*	*HSD3B7*	*NR1I3 (CAR)*		*CYP2D6*		*SLCO1B1*	*NR2B1 (RXRα)*
*ABCG8*	*SCP2*	*NR5A2 (LRH-1)*		*CYP2E1*		*SLCO1B3*	*NR3A1 (ERɑ)*
*ATP8B1*	*AKR1D1*	*NR2B1 (RXRα)*		*CYP3A4*		*SLCO2B1*	
*SLC10A1*	*SULT2A1*	*KL (Klotho β)*		*TPMT*		*ABCB1*	
*SLC10A2*	*UGT2B11*					*ABCC2*	
*SLCO1A2*						*ABCG2*	
*SLCO1B1*							
*SLCO1B3*							
*SLCO2B1*							
*SLC51A (OSTα)*							
*SLC52A (OSTβ)*							

**Fig. 2:**
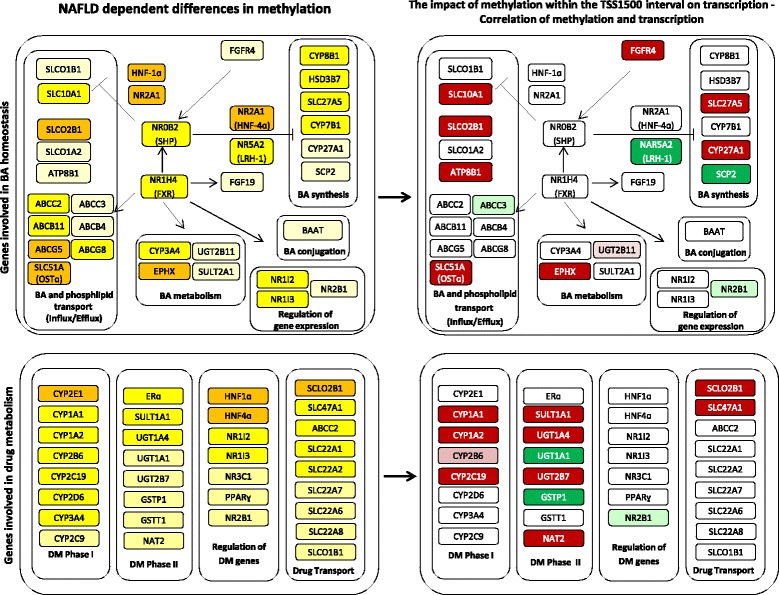
Schematic overview on genes displaying significant NAFLD dependent methylation changes in the TSS1500 interval and associated transcriptional changes. Genes involved in bile acid homeostasis are shown in the upper panel and genes involved in drug metabolism and disposition are shown in the lower panel. Color codes in the left panels indicate the strength of methylation changes in both gene clusters: category 1 (strong changes, dark yellow) defined as ≥ 50 % of CpG islands changed in genes with ≥7 CpG sites within the TSS1500 interval; category 2 (medium strong, yellow) defined as ≥50 % of CpG islands changed in genes with 3 to 6 CpG sites within the TSS1500 interval or 30–50 % of CpG islands changed in genes with ≥ 7 CpG sites); category 3 (small changes, light yellow) defined as < 3 CpG sites within the TSS1500 interval or ≥ 3 CpG islands within the TSS1500 interval and < 30 % of CpG islands changed in methylation. Color codes in the right panels indicate the direction of methylation change in dependency of fibrosis (red, hypermethylation, green, hypomethylation) and the direction of association between methylation and transcription (dark red and dark green, inverse association; light read and light green, parallel association). If no association between methylation and transcription was detected field were left white. The majority of methylation-transcription associations were inverse. Positive associations and correlations were observed for *ABCC3*, and *UGT2B11* (cluster 1), for *CYP2B6* (cluster 2) and for *NR2B1* (both clusters). *SLCO2B1* showed in the majority of cases fibrosis dependent hypermethylated CpG sites (6 of 9 nine sites, one hypomethylated site) and was therefore assigned to the hypermethylated targets

We further categorized the strength of NAFLD dependent methylation changes into three groups. Category 1 comprises genes with a high NAFLD dependent impact on methylation in combination with a high CpG site density, i.e. ≥ 7 CpG sites abundant within the TSS1500 interval, and ≥ 50 % of these sites significantly disease dependently changed in methylation (orange marked genes in Fig. 2). Category 2 includes genes carrying 3 to 6 CpG within the TSS1500 interval with ≥ 50 % of these sites significantly changed in methylation or carrying ≥ 7 CpG sites within the TSS1500 interval with 30–50 % of these sites significantly changed in methylation (yellow marked genes, Fig. 2). Category 3 includes all genes carrying < 3 CpG sites within the TSS1500 interval and genes with ≥ 3 CpG sites with < 30 % of these CpG sites affected in methylation (light yellow marked genes, Fig. 2).

Genes showing a high NAFLD dependent change in methylation combined with a high density of CpG sites within the TSS1500 interval (category 1) include the transporter genes *SLC51A* (OSTα), *ABCG5* and *SLCO2B1*, the nuclear receptors genes *NR2A1* (HNF4α) and *HNF1α* as well as *EPHX* in gene cluster 1 (6 of 43 genes, 13.9 %). Genes with a less strong CpG site density and a considerable significant disease dependent modulation in methylation (category 2) include the transporter genes *ABCB11* (BSEP), *SLC10A1* (NTCP), *ABCC2* (MRP2), the enzyme coding genes *CYP8B1*, *CYP3A4* and *CYP7B1* and the nuclear receptors *NR0B2* (SHP), *NR1I2*, *NR1I3* and *NR1H4*. The genes *NR5A2* (LRH-1), *SLC27A5* (BACS) and *CYP7B1* shall be mentioned within the group of genes only weakly affected in methylation (category 3), showing at least 4 CpG sites with altered methylation in NAFLD and a dense TSS1500 associated CpG site composition (≥12 CpG sites).

Fifteen of 41 genes (36.6 %) show considerable significant NAFLD associated modulations in methylation in gene cluster 2 (methylation category 2). This includes the genes encoding the drug metabolizing enzymes CYP2C9, CYP2C19, CYP3A4, CYP2D6, CYP1A2, CYP2C19 and UGT1A4 as well as the drug transporters MRP2 *(ABCC2)* and OAT2 (*SLC22A7*). In this category we also found *ERα*, *CYP1A1* and *SLC47A1* (MATE1) to be characterized by a very dense CpG site composition within the TSS1500 interval and to have at least 6 CpG sites associated with NAFLD-dependently altered methylation. Genes showing high NAFLD dependent modulation in methylation (category 1) comprise *CYP2E1* besides genes coding for HNF4α, HNF1α and CYP2B1, which have been also part of gene cluster 1 as a result of their overlapping functional role in both BA homeostasis and DM.

### NAFLD induced hepatic fibrosis is related to methylation changes in BA and DM associated genes

We validated the genes with significant NAFLD associated altered methylation profile within the TSS1500 interval of clusters 1 (BA homeostasis) and 2 (DM) using a cohort of 54 mild or advanced NAFLD patients (validation cohort, [13], E-GEOD-49542). Individuals had been characterized with regard to the severity of hepatic fibrosis in the frame of NAFLD. Multiple linear regression analyses were performed considering demographic data, the percentage of steatosis or stage of fibrosis as putative confounders.

As demonstrated in (Additional files 1: Table S1 and Additional file 2: Table S2 and in their extracts in Tables 3 and 4), the severity of liver fibrosis had a significant impact on the methylation of genes in both clusters. Strikingly, the severity of steatosis hardly impacted the epigenetic profile in both gene clusters, allowing the hypothesis that especially NAFLD associated fibrotic changes appear to be the driving force behind methylation shifts in both gene clusters. Interestingly, *ERɑ*, which is characterized by an exceptional high CpG site density within the TSS1500 interval shows epigenetic changes in dependency of both fibrotic and steatotic severity, thus, behaving in this respect discordant in comparison to the other gene targets investigated in gene cluster 1 and 2.

**Table 3:** Excerpt of NAFLD associated methylation changes in genes involved in bile acid homeostasis

					Discovery Group (*n* = 74)^b^			Validation Cohort (*n* = 54)^c^			
		CpG-sites			Healthy vs NAFLD&NASH			Fibrosis stage		Steatosis stage	
Gene	Total	Sign.	Sign. (%)^a^	Illumina ID	Coef.	*p*	*p* (FDR)	Coef.	*p*	Coef.	*P*
*UGT2B11*	1	1	100 %	cg27372994	3.94E-02	5.57E-04	2.63E-03	3.76E-02	6.80E-03	-	ns
*SLC51A*	7	6	86 %	cg17526770	4.49E-02	2.92E-04	2.07E-03	4.51E-02	4.23E-03	-	ns
				cg27558485	4.74E-02	2.82E-05	1.62E-03	4.88E-02	3.33E-03	-	ns
				cg08877188	3.19E-02	1.46E-03	5.18E-03	3.48E-02	1.81E-03	-	ns
				cg21748136	4.79E-02	2.31E-04	1.80E-03	4.60E-02	7.16E-03	-	ns
				cg05473677	3.17E-02	2.92E-03	8.21E-03	3.40E-02	8.50E-03	-	ns
				cg04478991	3.04E-02	4.23E-03	1.13E-02	3.48E-02	1.25E-02	-	ns
*EPHX1*	13	9	69 %	cg26187962	4.32E-02	3.61E-03	9.88E-03	5.34E-02	2.52E-03	-2.36E-02	2.66E-02
				cg03337430	6.13E-02	5.51E-04	2.63E-03	6.17E-02	1.08E-03	-	ns
				cg24928687	6.93E-02	5.95E-05	1.62E-03	7.78E-02	4.56E-05	-	ns
				cg17468616	6.00E-02	1.09E-04	1.62E-03	6.04E-02	1.50E-04	-	ns
				cg23096144	4.12E-02	4.80E-04	2.42E-03	4.09E-02	6.26E-04	-	ns
				cg03459809	3.77E-02	2.27E-03	7.00E-03	3.31E-02	2.73E-03	-	ns
				cg05385434	5.57E-02	2.04E-04	1.80E-03	6.29E-02	3.60E-03	-	ns
				cg25152404	4.71E-02	6.70E-05	1.62E-03	4.72E-02	2.97E-03	-	ns
				cg24868305	3.76E-02	9.58E-04	3.60E-03	4.47E-02	4.00E-03	-	ns
*SLCO2B1*	9	6	67 %	cg12537437	7.04E-02	9.27E-05	1.62E-03	8.53E-02	3.17E-05	-	ns
				cg25367084	6.36E-02	3.09E-04	2.11E-03	7.40E-02	4.99E-05	-	ns
				cg23577865	3.92E-02	7.32E-04	3.04E-03	4.27E-02	6.23E-04	-	ns
				cg20358275	3.49E-02	6.06E-03	1.55E-02	4.65E-02	5.81E-04	-	ns
				cg15751948	6.04E-02	1.06E-04	1.62E-03	4.73E-02	7.48E-03	-	ns
				cg18589858	-1.81E-02	6.54E-03	1.62E-02	-2.77E-02	3.31E-03	-	ns
*SLC10A1*	6	3	50 %	cg05633152	5.62E-02	2.12E-04	1.80E-03	5.59E-02	7.10E-04	-	ns
				cg21088438	4.80E-02	7.96E-04	3.25E-03	5.28E-02	3.38E-03	-	ns
				cg01448863	4.52E-02	1.33E-04	1.80E-03	3.56E-02	1.80E-02	-	ns
*SLC27A5*	13	5	38 %	cg19469742	3.92E-02	2.80E-03	8.17E-03	7.33E-02	3.81E-04	-	ns
				cg18495710	6.32E-02	3.31E-05	1.62E-03	5.19E-02	1.96E-03	-	ns
				cg16107172	5.07E-02	8.38E-05	1.62E-03	4.44E-02	4.82E-03	-	ns
				cg07726085	5.10E-02	1.65E-04	1.80E-03	3.75E-02	1.59E-02	-	ns
				cg16278661	3.88E-02	5.98E-04	2.67E-03	3.33E-02	1.77E-02	-	ns
				cg06621784	-4.72E-02	2.14E-03	6.81E-03	-8.29E-02	2.79E-06	-	ns
*ATP8B1*	9	2	22 %	cg12517027	-8.67E-02	5.29E-05	1.62E-03	-1.50E-01	1.28E-06	3.55E-02	3.90E-02
				cg21357291	3.93E-02	3.90E-04	2.14E-03	3.95E-02	1.58E-02	-	ns
*CYP27A1*	5	1	20 %	cg03460682	1.28E-02	1.69E-03	5.55E-03	3.15E-02	4.23E-03	-	ns
*NR5A2*	21	5	24 %	cg08158862	3.36E-02	1.16E-03	4.24E-03	4.73E-02	1.12E-03	-	ns
				cg16993043	2.08E-02	3.83E-03	1.04E-02	3.88E-02	4.29E-03	-	ns
				cg17804356	1.80E-02	4.07E-04	2.19E-03	2.68E-02	1.37E-02	-	ns
				cg21851672	-2.89E-02	3.89E-04	2.14E-03	-3.31E-02	4.85E-04	-	ns
				cg04308769	-5.82E-02	3.27E-04	2.11E-03	-4.56E-02	1.24E-03	-	ns
*FGFR4*	12	2	17 %	cg00618323	3.01E-02	1.23E-03	4.45E-03	2.65E-02	2.42E-03	-	ns
				cg04849878	5.45E-02	3.16E-04	2.11E-03	5.03E-02	3.88E-03	-	ns
*ABCC3*	8	1	13 %	cg27222669	-4.17E-02	1.07E-04	1.62E-03	-4.86E-02	1.36E-04	-	ns
*NR2B1*	9	1	11 %	cg14651936	-2.38E-02	2.95E-03	8.21E-03	-3.25E-02	5.67E-05	-	ns

The full results are shown in Additional file 1: Table S1. Shown here are only the genes, where methylation was later associated with transcriptional changes (see Table 5)

- no coefficient given for non significant results, *n* number, *ns*, not significant, *coef* coefficient, *FDR* false discovery rate, *sign* significant, *total* investigated in total

^a^Percentage of differentially methylated CpG sites in NAFLD within the TSS1500 interval as confirmed in two cohorts. Order of genes in the list follows the percentage of differentially methylated CpG sites within the TSS1500

^b^The discovery cohort is composed of liver healthy and NAFLD patients. Performance of multiple linear regression analyses investigating the association between methylation state of gene associated individuals CpG sites and hepatic disease state. Listed are raw *p*-values and false discovery (FDR) corrected p-values

^c^The validation cohort is composed of NAFLD patients with different stages of steatosis and fibrosis. Performance of multiple linear regression analyses investigating the association between methylation state of gene associated individuals CpG sites and severity of fibrosis and steatotis, respectively. Listed are raw *p*-values and FDR corrected *p*-values

**Table 4:** Excerpt of NAFLD associated methylation changes in genes involved in drug metabolism and disposition

					Discovery Group (*n* = 74)^b^			Validation Cohort (*n* = 54)^c^			
CpG-sites					Healthy vs NAFLD&NASH			Fibrosis stage		Steatosis stage	
Gene	Total	Sign.	Sign. (%)^a^	Illumina ID	Coef.	*p*	*p* (FDR)	Coef.	*p*	Coef.	*p*
*UGT1A1*	2	2	100 %	cg19146119	4.66E-02	8.71E-03	1.97E-02	8.02E-02	4.69E-04	-	ns
				cg07823755	-1.34E-02	1.18E-02	2.57E-02	-1.26E-02	1.41E-02	-	ns
*UGT1A4*	5	4	80 %	cg04437648	8.11E-02	5.65E-05	1.63E-03	8.83E-02	2.69E-05	-	ns
				cg05313279	5.12E-02	5.58E-03	1.37E-02	8.79E-02	1.07E-04	-	ns
				cg02234120	-3.48E-02	2.15E-04	1.93E-03	-3.02E-02	1.69E-03	-	ns
				cg05141602	-3.23E-02	3.00E-04	2.09E-03	-2.01E-02	4.28E-02	-	ns
*CYP1A2*	3	2	67 %	cg11473616	6.52E-02	6.09E-04	2.82E-03	7.37E-02	3.87E-04	-	ns
				cg04968473	5.22E-02	1.54E-02	3.22E-02	8.93E-02	8.76E-04	-	ns
*SLCO2B1*	9	6	67 %	cg12537437	7.04E-02	9.27E-05	1.63E-03	8.53E-02	3.17E-05	-	ns
				cg25367084	6.36E-02	3.09E-04	2.09E-03	7.40E-02	4.99E-05	-	ns
				cg23577865	3.92E-02	7.32E-04	3.21E-03	4.27E-02	6.23E-04	-	ns
				cg20358275	3.49E-02	6.06E-03	1.47E-02	4.65E-02	5.81E-04	-	ns
				cg15751948	6.04E-02	1.06E-04	1.64E-03	4.73E-02	7.48E-03	-	ns
				cg18589858	-1.81E-02	6.54E-03	1.53E-02	-2.77E-02	3.31E-03	-	ns
*ABCC2*	5	3	60 %	cg17044311	7.22E-02	1.01E-04	1.64E-03	7.84E-02	3.55E-04	-	ns
				cg14947634	7.21E-02	2.19E-04	1.93E-03	7.08E-02	9.22E-04	-	ns
				cg09448875	6.81E-02	1.87E-04	1.88E-03	6.60E-02	1.74E-03	-	ns
*CYP2C19*	6	3	50 %	cg00051662	8.55E-02	6.29E-03	1.49E-02	1.59E-01	1.06E-05	-	ns
				cg16227251	6.22E-02	5.35E-04	2.73E-03	7.07E-02	6.18E-03	-	ns
				cg04189838	7.22E-02	2.41E-02	4.95E-02	1.71E-01	1.02E-05	-	ns
*UGT2B7*	2	1	50 %	cg25583503	5.54E-02	3.16E-04	2.09E-03	6.47E-02	1.47E-03	-	ns
*SULT1A1*	7	3	43 %	cg01378222	6.76E-02	2.55E-04	1.98E-03	1.13E-01	1.55E-04	-	ns
				cg09685060	4.34E-02	3.10E-03	8.53E-03	5.18E-02	1.14E-04	-	ns
				cg08008286	5.22E-02	2.82E-04	2.03E-03	4.89E-02	2.73E-02	-	ns
*SLC47A1*	15	6	40 %	cg08895056	3.73E-02	3.82E-03	1.01E-02	7.57E-02	2.48E-05	-	ns
				cg10718608	2.85E-02	7.89E-03	1.80E-02	5.52E-02	4.48E-04	-	ns
				cg01530032	5.36E-02	9.66E-04	3.93E-03	1.08E-01	2.37E-06	-	ns
				cg12133118	3.28E-02	1.99E-03	6.36E-03	4.65E-02	4.65E-04	-	ns
				cg24151087	1.52E-02	1.86E-02	3.84E-02	2.03E-02	5.65E-03	-	ns
				cg15014549	6.87E-03	2.15E-03	6.76E-03	1.78E-02	2.40E-02	-	ns
*CYP2B6*	8	3	38 %	cg19756068	5.45E-02	1.20E-04	1.64E-03	4.51E-02	3.04E-03	-	ns
				cg10322876	4.75E-02	1.38E-03	5.10E-03	4.99E-02	1.79E-03	-	ns
				cg08852641	-5.57E-02	2.83E-05	1.63E-03	-5.09E-02	2.58E-04	-	ns
*CYP1A1*	17	6	35 %	cg12101586	6.63E-02	1.92E-05	1.63E-03	1.16E-01	1.62E-06	-	ns
				cg13570656	7.61E-02	3.93E-05	1.63E-03	1.45E-01	9.83E-06	-	ns
				cg00213123	5.41E-02	8.07E-05	1.63E-03	1.01E-01	7.50E-06	-	ns
				cg26516004	7.00E-02	8.42E-05	1.63E-03	1.41E-01	1.19E-06	-	ns
				cg17852385	6.24E-02	9.05E-05	1.63E-03	1.47E-01	9.82E-06	-	ns
				cg11924019	4.63E-02	4.57E-04	2.53E-03	9.71E-02	4.93E-05	-	ns
*NR2B1*	9	1	11 %	cg14651936	-2.38E-02	2.95E-03	8.23E-03	-3.25E-02	5.67E-05	-	ns
*GSTP1*	10	1	10 %	cg06928838	-5.34E-02	2.96E-03	8.23E-03	-8.12E-02	3.67E-04	-	ns

The full results are shown in (Additional file 1: Table S1). Shown here are only the genes, where methylation was later associated with transcriptional changes (see Table 6)

- no coefficient given for non significant results, *n* number, *ns* not significant, *coef* coefficient, *FDR* false discovery rate

^a^Percentage of differentially methylated CpG sites in NAFLD within the TSS1500 interval as confirmed in two cohorts. Order of genes in the list follows the percentage of differentially methylated CpG sites within the TSS1500

^b^The discovery cohort is composed of liver healthy and NAFLD patients. Performance of multiple linear regression analyses investigating the association between methylation state of gene associated individuals CpG sites and hepatic disease state. Listed are raw *p*-values and false discovery (FDR) corrected *p*-values

^c^The validation cohort is composed of NAFLD patients with different stages of steatosis and fibrosis. Performance of multiple linear regression analyses investigating the association between methylation state of gene associated individuals CpG sites and severity of fibrosis and steatotis, respectively. Listed are raw *p*-values and FDR corrected *p*-values

### CpG site methylation impacted by NAFLD significantly inversely correlates with the transcription of DM and BA genes and respective neighboring genes

To evaluate to what extent the methylation of CpG sites associated with NAFLD impacts the expression of cluster 1 and cluster 2 genes in healthy and diseased hepatic tissue, Pearson’s correlation analyses and robust linear regression analyses were performed in a cohort of 30 non-NAFLD and 20-NAFLD patients (expression correlation (EC) cohort, [13], E-GEOD-61258, Fig. 1, Table 1), aligning methylation state and level of transcriptional expression inraindividually to each other and taking the fibrosis stage into account. For these analyses genes were separated in disease dependently hyper- or hypomethylated targets, averaging the strength of differentially methylated sites to a representative value for the TSS1500 intervals, which were then investigated in context to transcription.

We found a significant association of methylation with transcription for 13 BA homeostasis associated genes (gene cluster 1), of which nine were hypermethylated and inversely correlated with transcription in NAFLD (Table 5). This includes the BA synthetizing and metabolizing genes *CYP27A1*, *EPHX* and *UGT2B11*, the BA transporter genes *SLC10A1* (also mentioned in [8]) and *SLCO2B1* as well as the genes *FGFR4* and *ATP8B1* involved in BA signaling and bile associated phospholipid transport. The BA transporting protein *ABCC3* and the nuclear receptor *NR2B1* were hypomethylated in NAFLD and displayed, in contrast to many other genes, a positive correlation of methylation and transcription. Methylation of 10 DM associated genes, i.e. the phase I DM genes *CYP1A1*, *CYP1A2*, *CYP2C19*, the phase II genes *SULT1A1*, *UGT1A1*, *UGT1A4*, *UGT2B7, GSTP1* and the transporter genes *SLC47A1*, *SLCO2B1* was detected to be significantly inversely associated with the transcriptional expression in gene cluster 2 (Table 6). With exception of *GSTP1*, *NR2B1* and *UGT1A1* all genes were hypermethylated in NAFLD. *CYP2B6* was detected to display a positive correlation between methylation state and transcription. It is worth noting that inverse correlations between methylation and transcription were especially observed in analyses including liver-healthy individuals and in robust linear regression analyses, taking the severity of steatosis and fibrosis into account. Unadjusted correlation analyses within the disease study arm of the EC cohort, which is composed of many individuals with simple steatosis and a low severity state of fibrosis, led only for a few targets to significant results.

**Table 5:** Methylation/transcription correlations of genes involved in bile acid homeostasis in NAFLD

						Pearson correlation analyses^d^			
	CpG-sites			Robust linear regression (*n* = 50)^b^		SS & NASH (*n* = 20)		Controls (*n* = 30)	
Gene	Total	Sign.^a^	*p* (binomial)	Coef.	*p*	Coef.	*p*	Coef.	*p*
*Hypermethylated targets*									
*ABCB11*	3	2	**7.25E-03**	-	*ns*	-	*ns*	-	*ns*
*ABCC2*	5	3	**1.16E-03**	-	*ns*	-	*ns*	-	*ns*
*ABCG5*	12	11	**5.59E-14**	-	*ns*	-	*ns*	-	*ns*
*ATP8B1*	9	1	*ns*	-	*ns*	-0.52	**1.77E-02**	-	*ns*
*CYP27A1*	5	1	*ns*	-0.70	**4.54E-05**	-	*ns*	-0.57	**9.50E-04**
*CYP7B1*	12	4	**2.24E-03**	-	*ns*	-	*ns*	-	*ns*
*CYP8B1*	6	5	**1.80E-06**	-	*ns*	-	*ns*	-	*ns*
***EPHX1***	13	9	**1.16E-09**	-0.50	**5.99E-03**	-	*ns*	-	*ns*
*FGFR4*	12	2	*ns*	-0.45	**8.05E-05**	-	*ns*	-0.39	**3.21E-02**
*HNF1A*	8	8	**3.91E-11**	-	*ns*	-	*ns*	-	*ns*
*NR0B2*	5	3	**1.16E-03**	-	*ns*	-	*ns*	-	*ns*
*NR1H4*	4	3	**4.81E-04**	-	*ns*	-	*ns*	-	*ns*
*NR1I3*	4	3	**4.81E-04**	-	*ns*	-	*ns*	-	*ns*
*NR2A1*	18	15	**2.16E-17**	-	*ns*	-	*ns*	-	*ns*
***SLC51A***	7	6	**1.05E-07**	-0.25	**2.37E-02**	-	*ns*	-	*ns*
***SLC10A1***	6	3	**2.23E-03**	-11.00	**3.67E-08** ^**c**^	-	*ns*	-	*ns*
***SLC27A5***	13	5	**2.87E-04**	-0.27	**1.42E-02**	-	*ns*	-	*ns*
***SLCO2B1***	9	5	**3.32E-05**	-0.38	**1.41E-03**	-	*ns*	-	*ns*
*UGT2B11*	1	1	*ns*	-	*ns*	0.56	**9.60E-03**	-	*ns*
*Hypomethylated targets*									
*ABCC3*	8	1	*ns*	-	*ns*	0.46	**3.94E-02**	-	*ns*
*CYP3A4*	3	2	**7.25E-03**	-	*ns*	-	*ns*	-	*ns*
*NR1I2*	5	2	**2.26E-02**	-	*ns*	-	*ns*	-	*ns*
*NR2B1*	9	1	*ns*	-	*ns*	-	*ns*	0.42	**2.09E-02**
*NR5A2*	21	2	*ns*	-	*ns*	-	*ns*	-0.44	**1.62E-02**
*SCP2*	7	1	*ns*	-	*ns*	-0.48	**3.15E-02**	-	*ns*
*SLCO2B1*	9	1	*ns*	0.26	**7.07E-03**	-	*ns*	0.47	**8.22E-03**

The Table lists genes with NAFLD dependent significant changes in the overall CpG site methylation within the TSS1500 interval after binomial testing and its association to transcriptional changes. Genes showing significant changes in average methylation with significant association to transcription are shown in bold

- no coefficient given for non significant results, *n* number, *ns* not significant, *coef* coefficient, *sign*. significant

^a^Number of significantly hyper- or hypomethylated CpG sites in discovery and validation cohort. Genes in the list appear according to their alphabetical order

^b^Regression analyses studying the association between average methylation in the TSS1500 interval and strength of transcription in the EC cohort. Analyses are taking the strength of fibrosis and steatosis into account

^c^Regression analyses under consideration of a fibrosis/expression interaction term

^d^Pearson’s correlation analyses correlating average methylation state in the TSS1500 interval and strength of transcription in NAFLD and non-NAFLD patients of the EC cohort

**Table 6:** Methylation/transcription correlations of genes involved in drug metabolism in NAFLD

						Pearson correlation analyses^c^			
	CpG-sites			Robust linear regression (*n* = 50)^b^		SS & NASH (*n* = 20)		Controls (*n* = 30)	
Gene	Total	sign.^a^	*p* (binomial)	Coef.	*p*	Coef.	*p*	Coef.	*p*
*Hypermethylated targets*									
*ABCC2*	5	3	**1.16E-03**	-	*ns*	-	*ns*	-	*ns*
***CYP1A1***	17	6	**1.20E-04**	-0.42	**3.83E-02** ^**c**^	-	*ns*	-	*ns*
***CYP1A2***	3	2	**7.25E-03**	-0.42	**3.47E-03**	-	*ns*	-	*ns*
***CYP2B6***	8	2	*ns*	0.41	**1.00E-03**	-	*ns*	0.43	**1.64E-02**
***CYP2C19***	6	3	**2.23E-03**	-0.44	**4.50E-05**	-	*ns*	-0.44	**1.43E-02**
*CYP2C9*	2	2	**2.50E-03**	-	*ns*	-	*ns*	-	*ns*
*CYP2D6*	3	2	**7.25E-03**	-	*ns*	-	*ns*	-	*ns*
*CYP2E1*	7	6	**1.05E-07**	-	*ns*	-	*ns*	-	*ns*
*HNF1A*	8	8	**3.91E-11**	-	*ns*	-	*ns*	-	*ns*
*NR2A1*	18	15	**2.16E-17**	-	*ns*	-	*ns*	-	*ns*
*NAT2*	2	1	*ns*	-0.26	**1.31E-02**	-	*ns*	-	*ns*
*NR1I3*	4	3	**4.81E-04**	-	*ns*	-	*ns*	-	*ns*
*SLC22A1*	3	3	**1.25E-04**	-	*ns*	-	*ns*	-	*ns*
*SLC22A7*	2	2	**2.50E-03**	-	*ns*	-	*ns*	-	*ns*
***SLC47A1***	15	6	**5.28E-05**	-0.34	**8.43E-05**	-	*ns*	-0.41	**2.31E-02**
***SLCO2B1***	9	5	**3.32E-05**	-0.38	**1.41E-03**	-	*ns*	-	*ns*
***SULT1A1***	7	3	**3.76E-03**	-0.42	**1.31E-04**	-	*ns*	-0.54	**2.15E-03**
***UGT1A4***	5	2	**2.26E-02**	-0.63	**4.52E-02**	-	*ns*	-	*ns*
***UGT2B7***	2	1	*ns*	-0.53	**4.01E-03**	-	*ns*	-0.43	**1.76E-02**
*Hypomethylated targets*									
*CYP3A4*	3	2	**7.25E-03**	-	*ns*	-	*ns*	-	*ns*
*GSTP1*	10	1	*ns*	-0.36	**2.43E-02**	-	*ns*	-	*ns*
*NR1I2*	5	2	**2.26E-02**	-	*ns*	-	*ns*	-	*ns*
*NR2B1*	9	1	*ns*	-	*ns*	-	*ns*	0.42	**2.09E-02**
*NR3A1*	47	20	**2.50E-14**	-	*ns*	-	*ns*	-	*ns*
*SLC22A2*	10	3	**1.15E-02**	-	*ns*	-	*ns*	-	*ns*
*SLCO2B1*	9	1	*ns*	0.26	**7.07E-03**	-	*ns*	0.47	**8.22E-03**
*UGT1A1*	2	1	*ns*	-	*ns*	-	*ns*	-0.36	**4.90E-02**
*UGT1A4*	5	2	**2.26E-02**	-	*ns*	-	*ns*	-	*ns*

The Table lists genes with NAFLD dependent significant changes in the overall CpG site methylation within the TSS1500 interval after binomial testing and its association to transcriptional changes. Genes showing significant changes in average methylation with significant association to transcription are shown in bold

- no coefficient given for non significant results, *n* number, *ns* not significant, *coef* coefficient, *sign.* significant

^a^Number of significantly hyper- or hypomethylated CpG sites. Genes in the list appear according to their alphabetical order

^b^Regression analyses investigating the association between average methylation in the TSS1500 interval and strength of transcription in the EC cohort. The analyses are taking the strength of fibrosis into account

^c^Pearson’s correlation analyses correlating average methylation state in the TSS1500 interval and strength of transcription in NAFLD and non-NAFLD patients of the EC cohort

Several genes did not show any significant methylation-transcription associations despite a high CpG site density and a significant variability of methylation observed in NAFLD. This includes the NAFLD dependently hypomethylated gene *ERɑ*, and the NAFLD-dependently hypermethylated genes *ABCG5, HNF-1α* and *HNF-4α*.

To further validate our findings we investigated to what extent differences in CpG site methylation of those genes, significantly changed in expression and listed in Tables 5 and 6, affect the expression of five neighboring genes downstream and upstream of the respective gene targets in gene cluster 1 and 2. As demonstrated in (Additional file 5: Table S5), NAFLD associated differences in methylation of genes in cluster 1 and 2 had in many cases a significant impact on the expression of neighboring genes as well. The strongest associations between CpG site methylation and gene expression of neighboring genes were observed for the regulatory regions of the genes *EPHX* and *FGFR4* in gene cluster 1 and of *CYP1A2* in gene cluster 2, showing an association with transcriptional changes of more than 3 neighboring genes. These findings further underline the strong impact of NAFLD associated differences in methylation on regulation of especially those genes.

## Discussion

In a robust study performing a systematic and targeted analysis in three independent cohorts of NAFLD patients we demonstrate for the first time that NAFLD is specifically related to significant changes in the hepatic methylation of genes responsible for synthesis, transport and metabolism of BAs, in genes responsible for drug metabolism and transport and in genes regulating the transcription of these gene clusters. We show that the detection of methylation changes at CpG sites within the adjacent 1500 bp interval up- and downstream of the TSS are valuable surrogate markers for functionally relevant epigenetic shifts observed in NAFLD in the investigated gene networks.

Several studies show that the interplay of BAs and FXR leads to a reduction of triglyceride levels in plasma and inhibits hepatic fat accumulation [21, 22]. BA dependent activation of FXR furthermore inhibits hepatic gluconeogenesis and peripheral insulin sensitivity, which is associated with lower plasma glucose levels as demonstrated in several animal studies [23, 24]. We highlight and add especially OSTα, *SLCO2B1*, *EPHX, SLC27A5* and *CYP27A1,* besides *SLCO10A1* [8], to the list of BA homeostasis genes which show disease dependent methylation pattern changes in proximity to the TSS that impacts on transcriptional expression. An interesting observation is, that FXR and other nuclear receptors targeted by BAs are not significantly differentially methylated in any stages of NAFLD, allowing the hypothesis that epigenetic changes of BA and DM genes are especially in progressed NAFLD a major regulatory mechanism for the transcriptional regulation of these genes. The observation that especially fibrosis is strongly associated with methylation shifts of functional relevance in the BA gene network suggests that especially patients suffering from NASH may show changes in BA composition and associated consequences for lipid and glucose homeostasis. These observations are well in line with study results published by Murphy et al. and Ahrens et al., who both observed genome wide methylation shifts especially in patients showing NASH associated fibrosis [8, 13]. Recently, Lake et al. compared the BA composition and the transcriptomic profile of genes important for BA homeostasis in NAFLD patients and liver healthy controls. The authors showed that NAFLD leads to significant changes in the plasma BA profile, which appears to be based on an activation of the CYP7B1 dependent alternative BA synthesis pathway, putatively induced by expression changes of BA synthesizing enzymes, such as CYP8B1, CYP7B1 and CYP27A1 [19]. The NAFLD related hypermethylation in CYP27A1, as observed in our study, may be at least partly responsible for the expression changes seen for CYP27A1 in NAFLD. An inverse methylation-transcription behavior was, besides for NTCP (also mentioned in [8]), also observed for the hepatic uptake transporter SLCO2B1 and the efflux transporter OSTα. These observations indicate that the transport of BA in the hepatocyte and from the hepatocyte into the canalicular system might be modulated in NAFLD. Recently, it was demonstrated in vivo that the concentration of circulating plasma BA increases with progression of NAFLD [25]. It would be interesting to test in a future study whether the observed NAFLD related epigenetic changes are partly responsible for these in vivo effects.

We detected important genes responsible for phase I (*CYP1A1*, *CYP1A2*, *CYP2B6*, besides *CYP2C19* [8]), phase II (*GSTP1*, *SULT1A1*, *SULT1A4*, *UGT2B7*) and phase III (*SLC47A1*) to show NAFLD dependent methylation changes that are associated with inverse transcriptional changes. These findings point to consequences for the individual safety and efficacy with therapeutics that are substrates for these proteins in NAFLD patients. Similar to genes involved in BA homeostasis, methylation changes in DM genes are especially observed in fibrotic stages of NAFLD. We hypothesize that additional modulating effects on DM in NAFLD are induced by NAFLD associated shifts in BA composition and concentration that have consecutive effects on NR dependent regulatory pathways. NAFLD dependent pharmacokinetic studies investigating the in vivo behavior of therapeutics linked to the detected differentially methylated enzymes and transporters have not yet been performed to our knowledge. Those studies would be essential to further evaluate the importance of the here observed disease dependent methylation-transcription changes in DM enzymes and drug transporters.

For several genes, such as e.g. *HNF1ɑ, HNF4α, ABCG5* or *NR3A1* (ERɑ)*,* we did not observe any significant correlations between the strength in methylation and mRNA levels, despite the fact that fibrosis dependent changes in the methylation pattern were observed. These genes are linked to a high number of transcript identifiers on the Affymetrix gene expression chip, which may result in an unspecific detection signal for the respective mRNAs. Therefore we do not want exclude that NAFLD dependent modulations in the methylation of these genes have an impact on the expression of these genes as well.

Our study combines samples and data from three different data sets, which allowed for robust detection of NAFLD dependent methylation shifts in the gene clusters in focus as a basis for subsequent methylation-transcription correlations. We took CpG sites within the TSS1500 into account as it can be assumed that methylation changes within these regions may especially be able to induce changes in the expression of adjacent genes. We defined and validated an epigenetic marker for the methylation state of significantly changed genes based on the average methylation within the TSS interval. This procedure is of great value for a robust epigenetic characterization of a gene. It can, however, not be excluded, that CpG sites further distal from the TSS may have as well an impact on the expression of genes investigated in the frame of this study.

There are known epigenetic changes in the liver due to changes in lipid and glucose metabolism as well as DNA damage and repair, fibrosis and liver tissue remodeling. Previous comprehensive genome-wide methylation analyses found changes in e.g. PGC1α, a key transcriptional regulator of mitochondrial fatty acid oxidation, has been associated with insulin resistance in NAFLD patients [8, 26]. There has not been, however, a comprehensive assessment performed of the association of NAFLD with or without changes in lipids or glucose levels and epigenetic changes in genes associated to bile acid or drug metabolism. We, thus, decided to take the known factors impacting strongly on bile acid and drug metabolism into consideration that were available in all three cohorts, i.e. gender, BMI and age. It may be of value to scrutinize in future studies to what extent variations in lipid composition and glucose homeostasis further induce epigenetic shifts in the gene networks investigated. Furthermore it will be important in the future to study the relation of methylation and transcription in larger cohorts as soon as available, comprising a larger fraction of patients suffering from NASH associated fibrosis, as an epigenetic changes in CpG sites significantly associated with transcription were especially observed in this patient group.

## Conclusions

We were able to demonstrate that the transcriptional expression of genes belonging to the closely related gene networks regulating BA homeostasis and DM is strongly coupled to epigenetic patterns near the transcriptional start site that are significantly shifting in patients with NASH associated hepatic fibrosis. These findings add further valuable information on how shifts in the BA profile and BA driven signaling pathways important for lipid and glucose homeostasis may arise in NAFLD patients and underline the importance for pharmacokinetic studies in patients suffering from NAFLD.

## Methods

### Cohorts

Our study includes data from three independent, published cohorts (Array Express Database, http://www.ebi.ac.uk/arrayexpress/) obtained from patients with NAFLD [8, 13, 27]. Ahrens et al. studied NAFLD in a case–control fashion and collected samples from patients who underwent liver biopsy for suspected NAFLD before and 5 to 9 months after bariatric surgery, showing the whole spectrum of liver histology, i.e. normal hepatic tissue, steatosis or NAFLD/NASH. Furthermore, Ahrens et al. collected 18 non-NAFLD samples (normal controls) from individuals undergoing major oncological surgery who underwent liver biopsy to exclude any hepatic malignant processes. Ahrens and co-workers provided methylation data of altogether 85 individuals under E-GEOD-48325. All 29 NAFLD patients (14 SS and 15 NASH) and all 45 non-NAFLD patients (18 normal controls plus 27 healthy obese individuals) were integrated as discovery cohort in our analysis (E-GEOD-48325, 11 individuals are specified as “diagnosis not known” in this dataset and were, thus, not included in our analysis, Fig. 1). Participants from Murphy et al. [13] served as validation cohort in our investigation. Murphy et al. correlated whole genome wide methylation of NAFLD patients with changes in the whole transcriptome by the severity of fibrosis. Methylation levels, stage of fibrosis and percentage steatosis of 54 patients were included in our analysis (validation cohort, E-GEOD-49542). Demographic information such as BMI and age, fibrosis stage and percent steatosis was available for all patients. Horvath et al. [27] studied changes on the methylation and transcriptional level in an epigenetic biomarker for aging in liver tissue of NAFLD patients on the basis of data published by Ahrens et al. [8] and validated their results in additionally collected liver samples from NAFLD and non-NAFLD patients. Matching methylation and transcriptional data of 45 control patients and 20 NAFLD patients were included in our analysis (expression correlation (EC) cohort, E-GEOD-61258). Details on demographic and clinical data of the patients included in our analysis and on sample size and composition of each cohort are summarized in Table 1 and Fig. 1.

Data and material of the study cohort at Duke University were collected as part of the Duke University Health System NAFLD Clinical Database and Biorepository. This biorepository is approved by the Institutional Review Board at Duke University and contains clinical data, serum, plasma, and frozen liver tissue from NAFLD patients who underwent diagnostic liver biopsy to grade and stage severity of disease as part of standard of care. Biospecimens are collected at the time of liver biopsy and after a 12 h fast for the scheduled procedure. Only patients who consented to utilize their samples for “-omics” analysis were included in the analysis. The cohort study at University of Kiel was approved by the institutional review board (“Ethikkommission der Medizinischen Fakultät der Universität Kiel,” D425/07, A111/99) before the commencement of the study and all patients provided written, informed consent.

### Disease staging of NAFLD

Information about the fibrotic severity, subdivided into the states “insignificant”, “mild” or “advanced” in the validation cohort and categorized in the stages 0, 0.5, 1, 2 in the EC cohort, were available and matched to each other (‘Mild’ (1), ‘Advanced’ (2), and ‘Insignificant’ (0 or 0.5)). Diagnostic criteria for fibrotic staging have been described in detail in [8, 13, 27]. The percent steatosis in each of the liver samples was divided into four levels: <5 %, 5–33 %, 34–66 % and >66 % in the EC cohort and matched to the strength of steatosis, defined as levels 0 to 3, in the validation cohort (0 = ' < 5 %', 1 = '5-33 %', 2 = '34–66 %' and 3 = ' > 66 %').

### Microarray analysis

The genome wide methylation profiles of liver samples published by Ahrens et al., Murphy et al. and Horvath et al. [8, 13, 27] were generated by hybridizing bisulfite converted DNA on the Illumina HumanMethylation450 Beadchip (Illumina, SanDiego, CA). Expression data in the study of Horvath et al. [27] were obtained using the HuGene 1.1 STGene expression array (Affymetrix Inc., Santa Clara, CA). In both studies DNA and RNA was extracted from frozen liver tissue using extraction kits from Qiagen.

### Gene annotation and probe selection

For probe set annotation in the Illumina Infinium HumanMethylation450 Beadchip array, we used the expanded annotation table provided by Price et al. [28]. This file was used for associating gene and transcript with respective CpG site, the distance to the closest transcription start site and the occurrence of any single nucleotide polymorphism (SNP) loci in the CpG site. CpG sites were excluded from the analysis if carrying a known SNP locus or if located outside the area 1500 bp upstream or downstream from the transcriptional start site (TSS1500 interval). The probe set annotation file provided by Affymetrix (Affymetrix Inc., Santa Clara, CA) was used to associate a gene transcript with probes on the Affymetrix chip. On this array, probes may be annotated to more than one individual transcript.

### Gene Set enrichment analysis

We used the scientific literature and the databases of the Gene Ontology (GO) enrichment consortium (http://geneontology.org/page/go-enrichment-analysis) and KEGG (http://www.genome.jp/kegg/) to compile two gene clusters for the analysis of methylation and transcription data; one specifically involved in BA synthesis, transport and metabolism and the second focused on drug metabolism, transport and disposition. In this frame key words including “bile acid metabolism”, “bile acid synthesis”, “bile acid transport”, “drug metabolism”, and “drug transport” were used in the programs to detect the relevant genes of interest. The BA associated gene cluster (gene cluster 1) comprised 43 genes and covered, besides genes encoding hepatic transporters and enzymes, genes expressing important members of the nuclear receptor family, such as FXR and PXR that are targeted by BAs. The drug metabolism associated gene cluster (gene cluster 2) comprised 41 genes including phase I and phase II metabolic enzymes, transport proteins and important nuclear receptors involved in gene transcription. Several genes are involved in both processes (e.g. PXR, OATP1B1) and have, thus, been considered in the analyses of both clusters. Table 2 summarizes the analyzed genes included in the two clusters.

### Statistical analysis

All downstream analyses were performed using R statistical packages (www.r-project.org, R version 3.1.2)*.* We performed the analytical steps described separately for each of the two investigated gene clusters. First, multiple linear regression was used to detect NAFLD dependent differences in the methylation state of CpG sites within the regulatory regions of the genes of interest in the discovery cohort. During this step, β-values of individual CpG sites of the candidate genes were separately evaluated. Age, gender, BMI, and disease state were considered as putative confounders. False discovery rate (FDR) corrected *p* values < 0.05 were considered as significant. Significantly differentially methylated CpG sites detected in the discovery cohort were then validated in the validation cohort. Here, the relationship between methylation β-values and disease progression, (i.e., percent steatosis and fibrosis stage) were again investigated via multiple linear regression analysis, controlling for age, gender and BMI. In this targeted analysis unadjusted *p* values < 0.05 were considered as significant.

CpG sites consistently significantly hypo- or hypermethylated in discovery and validation cohort were further investigated with regard to their association with transcriptional expression of the respective gene in the EC cohort. In a post validation step, we performed binomial tests to investigate if a gene had a statistically significant overrepresentation of differentially methylated CpG-sites within the TSS1500 interval, contrasting, for each gene separately, the proportion of significant probes to the total number probes at a *p*-value limit of 0.05 (Tables 3 and 4). In the next step, methylation and expression levels of each gene were correlated intra-individually. After subdividing CpG sites into hypo- and hypermethylated loci β-values within the TSS1500 interval (the up- and downstream located 1500 bp interval adjacent to the transcriptional start site) were averaged for each gene in the EC cohort. To reach a better conformity with expression levels, mean methylation β-values were transformed into M-values, i.e. the log_2_ transformed intensity ratios of methylated probe to unmethylated probe [29]. Robust linear regression analyses were performed intraindividually correlating averaged methylation values to expression levels adjusting for fibrosis alone and for an interaction term composed of fibrosis and expression, respectively. For these analyses we applied the R package “robust” and the R function “lmRob”, which performs a robust linear regression with high breakdown point and high efficiency regression. In a sub analysis we performed uncorrected Pearson’s correlation analyses correlating methylation with expression in a subset of liver diseased and non-diseased patients in the EC cohort. *P*-values < 0.05 were considered significant.

## Abbreviations

ABCC2, ATP-binding cassette C2; ABCG5, ATP-binding cassette G5; AhR, arylhydrocarbon receptor; BA, bile acids; BAAT, bile acid CoA amino acid N-acyltransferase; BACS, bile acid CoA synthetase; bp, base pair(s); BSEP, bile salt export pump; CYP, cytochrome P450; DM, drug metabolism; EC cohort, expression correlation cohort; EPHX, microsomal epoxide hydrolase; ER, estrogen receptor; FDR, false discovery rate; FGF(R), fibroblast growth factor (receptor); FXR, farnesoid X receptor; GR, glucocorticoid receptor; GST, glutathione transferase; HNF, hepatocyte nuclear factor; HSD3B7, hydroxy-delta-5-steroid-dehydrogenase; KL, Klothoβ; LRH-1, liver receptor homolog-1; MATE1, multidrug and toxin extrusion protein; MRP2, multidrug resistance-associated protein 2; NAFLD, non-alcoholic fatty liver disease; NASH, non-alcoholic steatohepatitis; NR, nucleotide receptor; NTCP, natrium taurocholate co-transporting polypeptide; OAT2, organic anion transporter 2; OSTα, organic solute transporter α; PGC1α, co-activator 1α; PXR, pregnane X receptor; RXR, retinoid X receptor; SCP2, sterol carrier protein 2; SHP, small heterodimer partner; SLC, solute carrier; SNP, single nucleotide polymorphism; SS, steatosis; SULT, sulfotransferase; TSS1500 interval, the adjacent 1500 bp up- and downstream from the transcription start site; UGT, UDP-glucuronosyltransferase

## Additional files

Additional file 1: Table S1. NAFLD associated methylation changes in genes involved in bile acid homeostasis – significant findings in gene cluster 1 (bile acid associated gene cluster). (PDF 184 kb) Additional file 2: Table S2. NAFLD associated methylation changes in genes involved in drug metabolism – significant findings in gene cluster 2 (genes involved in drug transport and metabolism). (PDF 183 kb) Additional file 3: Table S3. Methylation changes in genes involved in bile acid homeostasis (gene cluster 1) – percent methylation of significantly changed CpG sites in healthy individuals and in patients with different stages of NAFLD. (PDF 204 kb) Additional file 4: Table S4. Methylation changes in genes involved in drug transport and metabolisms – percent methylation of significantly changed CpG sites in healthy individuals and in patients with different stages of NAFLD. (PDF 182 kb) Additional file 5: Table S5. NAFLD dependent associations between the methylation of CpG sites belonging to genes listed in Table 5 and 6 and transcripts of a maximum of 10 neighboring genes. (PDF 226 kb)
