# Supplementary material for: A targeted analysis reveals relevant shifts in the methylation and transcription of genes responsible for bile acid homeostasis and drug metabolism in non-alcoholic fatty liver disease
Source: BMC Genomics. 2016 Jun 14;17:462. doi: 10.1186/s12864-016-2814-z (PMC4908840; doi:10.1186/s12864-016-2814-z)
Supplement: Additional file 1: Table S1. — NAFLD associated methylation changes in genes involved in bile acid homeostasis – significant findings in gene cluster 1 (bile acid associated gene cluster). (PDF 184 kb) [file 12864_2016_2814_MOESM1_ESM.pdf]

A targeted analysis reveals relevant shifts in the methylation and transcription of genes responsible for bile acid homeostasis and drug metabolism in non-alcoholic fatty liver disease

Supplement

Authors: H. B. Schiöth[1], A. Boström[1], S. K. Murphy[2], W. Erhart[3], J. Hampe[4], C. Moylan[5,6] and J. Mwinyi[1]

[1]Department of Neuroscience, Functional Pharmacology, Uppsala University, Uppsala, Sweden

[2]Department of Obstetrics and Gynecology, Duke University Medical Center, Durham, North Carolina, USA

[3]Department of Internal Medicine I, University Hospital Schleswig-Holstein, Kiel, Germany

[4]Medical Department I, University Hospital Dresden, Dresden, Germany

[5]Department of Medicine, Duke University Medical Center, Durham, North Carolina, USA

[6]Department of Medicine, Durham Veterans Affairs Medical Center, Durham, North Carolina, USA

Additional File 1: Table S1. NAFLD associated methylation changes in genes involved in bile acid homeostasis – significant findings in gene cluster 1 (bile acid associated gene cluster)

Table S1 NAFLD associated methylation changes in genes involved in bile acid homeostasis – significant findings in gene cluster 1

					Discovery Group (n=74)**			Validation Cohort (n=54)***			
	CpG-sites				Healthy vs NAFLD&NASH			Fibrosis stage		Steatosis stage	
Gene	Total	sign.	sign. (%)*	Illumina ID	Coef.	*p*	*p* (FDR)	Coef.	*p*	Coef.	*P*
ABCG8	1	1	100%	cg00760272	7.35E-02	**1.67E-04**	**1.80E-03**	7.75E-02	3.36E-04	-	ns
UGT2B11	1	1	100%	cg27372994	3.94E-02	**5.57E-04**	**2.63E-03**	3.76E-02	6.80E-03	-	ns
ABCG5	12	11	92%	cg01186613	7.35E-02	**2.25E-04**	**1.80E-03**	7.65E-02	1.16E-04	-	ns
				cg00009421	4.42E-02	**2.24E-04**	**1.80E-03**	4.55E-02	2.63E-03	-	ns
				cg07681696	5.79E-02	**9.42E-05**	**1.62E-03**	5.39E-02	2.46E-03	-	ns
				cg16451365	4.67E-02	**3.90E-04**	**2.14E-03**	4.59E-02	3.57E-03	-	ns
				cg11467440	3.33E-02	**2.02E-03**	**6.56E-03**	3.43E-02	2.56E-03	-	ns
				cg18281102	5.42E-02	**9.33E-05**	**1.62E-03**	4.65E-02	6.00E-03	-	ns
				cg25781162	4.74E-02	**4.60E-05**	**1.62E-03**	4.45E-02	9.33E-03	-	ns
				cg00705576	3.83E-02	**3.80E-04**	**2.14E-03**	3.85E-02	7.20E-03	-	ns
				cg11113753	4.44E-02	**7.39E-05**	**1.62E-03**	3.40E-02	7.68E-03	-	ns
				cg08453096	4.58E-02	**2.35E-03**	**7.05E-03**	4.35E-02	1.47E-02	-	ns
				cg13341470	3.65E-02	**1.01E-03**	**3.77E-03**	2.99E-02	1.56E-02	-	ns
HNF1A	8	8	100%	cg01341572	4.22E-02	**5.69E-04**	**2.65E-03**	5.44E-02	1.69E-03	-	ns
				cg05934698	3.67E-02	**1.33E-03**	**4.78E-03**	5.36E-02	1.93E-03	-	ns
				cg25477769	3.19E-02	**3.37E-04**	**2.11E-03**	3.60E-02	1.87E-03	-	ns
				cg23661013	5.16E-02	**2.67E-04**	**2.03E-03**	4.41E-02	3.53E-03	-	ns
				cg02153339	4.83E-02	**6.60E-04**	**2.83E-03**	4.70E-02	4.50E-03	-	ns
				cg01394199	2.28E-02	**1.19E-02**	**2.58E-02**	4.39E-02	4.29E-04	-	ns
				cg14996810	5.13E-02	**3.39E-04**	**2.11E-03**	4.59E-02	1.95E-02	-	ns
				cg06444781	3.20E-02	**1.50E-03**	**5.20E-03**	3.94E-02	2.04E-02	-	ns
OSTalpha	7	6	86%	cg17526770	4.49E-02	**2.92E-04**	**2.07E-03**	4.51E-02	4.23E-03	-	ns
				cg27558485	4.74E-02	**2.82E-05**	**1.62E-03**	4.88E-02	3.33E-03	-	ns
				cg08877188	3.19E-02	**1.46E-03**	**5.18E-03**	3.48E-02	1.81E-03	-	ns
				cg21748136	4.79E-02	**2.31E-04**	**1.80E-03**	4.60E-02	7.16E-03	-	ns
				cg05473677	3.17E-02	**2.92E-03**	**8.21E-03**	3.40E-02	8.50E-03	-	ns
				cg04478991	3.04E-02	**4.23E-03**	**1.13E-02**	3.48E-02	1.25E-02	-	ns
CYP8B1	6	5	83%	cg21979032	4.98E-02	**1.87E-04**	**1.80E-03**	4.99E-02	6.24E-04	-	ns
				cg20365867	6.75E-02	**3.92E-05**	**1.62E-03**	6.35E-02	5.76E-04	-	ns
				cg26124860	6.02E-02	**4.53E-04**	**2.39E-03**	5.76E-02	4.33E-03	-	ns
				cg07147573	4.66E-02	**1.50E-03**	**5.20E-03**	4.94E-02	6.18E-03	-	ns
				cg14404298	3.72E-02	**2.93E-03**	**8.21E-03**	3.02E-02	4.34E-02	-	ns
NR2A1	18	15	83%	cg27420224	5.55E-02	**8.33E-04**	**3.30E-03**	6.45E-02	2.09E-04	-	ns
				cg23792485	5.47E-02	**5.16E-04**	**2.56E-03**	6.02E-02	8.44E-04	-	ns
				cg06640637	5.00E-02	**6.27E-04**	**2.76E-03**	5.82E-02	1.07E-03	-	ns
				cg20848979	4.53E-02	**3.75E-04**	**2.15E-03**	4.82E-02	9.45E-04	-	ns
				cg08314996	4.99E-02	**8.35E-04**	**3.30E-03**	4.94E-02	2.97E-03	-	ns
				cg21081369	2.95E-02	**4.01E-03**	**1.08E-02**	3.83E-02	3.38E-03	-	ns
				cg19717150	3.37E-02	**6.20E-03**	**1.57E-02**	4.21E-02	4.00E-04	-	ns

				cg07006673	4.31E-02	**5.52E-03**	**1.45E-02**	5.12E-02	3.50E-03	-	ns
				cg23834593	3.91E-02	**2.19E-03**	**6.89E-03**	3.47E-02	7.91E-03	-	ns
				cg03862380	4.20E-02	**3.68E-04**	**2.14E-03**	4.07E-02	6.72E-03	-	ns
				cg16221969	3.28E-02	**1.63E-03**	**5.52E-03**	3.19E-02	2.42E-02	-	ns
				cg21559386	4.57E-02	**5.99E-04**	**2.67E-03**	2.97E-02	3.58E-02	-	ns
				cg06126829	4.47E-02	**3.68E-04**	**2.14E-03**	3.47E-02	2.66E-02	-	ns
				cg20265805	3.12E-02	**2.46E-03**	**7.23E-03**	2.31E-02	4.60E-02	-	ns
				cg24084358	3.90E-02	**1.63E-03**	**5.52E-03**	3.46E-02	3.73E-02	-	ns
NR1I3	4	3	75%	cg06277277	5.23E-02	**1.55E-04**	**1.80E-03**	4.51E-02	2.91E-03	-	ns
				cg25214346	4.26E-02	**1.28E-04**	**1.80E-03**	3.49E-02	1.38E-02	-	ns
				cg17028814	4.53E-02	**8.58E-04**	**3.35E-03**	3.84E-02	1.19E-02	-	ns
NR1H4	4	3	75%	cg01202639	6.05E-02	**6.78E-05**	**1.62E-03**	3.81E-02	2.37E-02	-	ns
				cg08098450	6.53E-02	**9.44E-04**	**3.59E-03**	6.90E-02	1.12E-03	-	ns
				cg03771004	5.75E-02	**2.84E-04**	**2.06E-03**	4.07E-02	3.83E-02	-	ns
EPHX1	13	9	69%	cg26187962	4.32E-02	**3.61E-03**	**9.88E-03**	5.34E-02	2.52E-03	-2.36E-02	2.66E-02
				cg03337430	6.13E-02	**5.51E-04**	**2.63E-03**	6.17E-02	1.08E-03	-	ns
				cg24928687	6.93E-02	**5.95E-05**	**1.62E-03**	7.78E-02	4.56E-05	-	ns
				cg17468616	6.00E-02	**1.09E-04**	**1.62E-03**	6.04E-02	1.50E-04	-	ns
				cg23096144	4.12E-02	**4.80E-04**	**2.42E-03**	4.09E-02	6.26E-04	-	ns
				cg03459809	3.77E-02	**2.27E-03**	**7.00E-03**	3.31E-02	2.73E-03	-	ns
				cg05385434	5.57E-02	**2.04E-04**	**1.80E-03**	6.29E-02	3.60E-03	-	ns
				cg25152404	4.71E-02	**6.70E-05**	**1.62E-03**	4.72E-02	2.97E-03	-	ns
				cg24868305	3.76E-02	**9.58E-04**	**3.60E-03**	4.47E-02	4.00E-03	-	ns
ABCB11	3	2	67%	cg20118424	6.94E-02	**1.46E-04**	**1.80E-03**	6.96E-02	1.82E-03	-	ns
				cg00295325	4.27E-02	**2.07E-03**	**6.66E-03**	4.40E-02	7.17E-03	-	ns
CYP3A4	3	2	67%	cg23326197	-3.69E-02	**1.80E-05**	**1.62E-03**	-3.23E-02	2.36E-04	-	ns
				cg22821554	-1.98E-02	**1.68E-03**	**5.55E-03**	-2.50E-02	9.33E-04	-	ns
SLCO2B1	9	6	67%	cg12537437	7.04E-02	**9.27E-05**	**1.62E-03**	8.53E-02	3.17E-05	-	ns
				cg25367084	6.36E-02	**3.09E-04**	**2.11E-03**	7.40E-02	4.99E-05	-	ns
				cg23577865	3.92E-02	**7.32E-04**	**3.04E-03**	4.27E-02	6.23E-04	-	ns
				cg20358275	3.49E-02	**6.06E-03**	**1.55E-02**	4.65E-02	5.81E-04	-	ns
				cg15751948	6.04E-02	**1.06E-04**	**1.62E-03**	4.73E-02	7.48E-03	-	ns
				cg18589858	-1.81E-02	**6.54E-03**	**1.62E-02**	-2.77E-02	3.31E-03	-	ns
ABCC2	5	3	60%	cg17044311	7.22E-02	**1.01E-04**	**1.62E-03**	7.84E-02	3.55E-04	-	ns
				cg14947634	7.21E-02	**2.19E-04**	**1.80E-03**	7.08E-02	9.22E-04	-	ns
				cg09448875	6.81E-02	**1.87E-04**	**1.80E-03**	6.60E-02	1.74E-03	-	ns
NR0B2	5	3	60%	cg16177693	4.36E-02	**5.89E-04**	**2.67E-03**	4.86E-02	9.61E-04	-	ns
				cg06650260	4.87E-02	**8.62E-05**	**1.62E-03**	5.18E-02	2.27E-03	-	ns
				cg16762386	4.85E-02	**3.23E-04**	**2.11E-03**	4.03E-02	1.29E-02	-	ns
NR1I2	5	3	60%	cg22276685	5.57E-02	**7.82E-05**	**1.62E-03**	5.25E-02	2.54E-03	-	ns
				cg00836482	-3.51E-02	**9.22E-05**	**1.62E-03**	-3.42E-02	1.56E-03	-	ns
				cg25562664	-2.51E-02	**2.43E-03**	**7.21E-03**	-5.21E-02	7.57E-05	-	ns
BAAT	2	1	50%	cg05770171	6.60E-02	**4.79E-04**	**2.42E-03**	6.65E-02	1.38E-03	-	ns
SLC10A1	6	3	50%	cg05633152	5.62E-02	**2.12E-04**	**1.80E-03**	5.59E-02	7.10E-04	-	ns
				cg21088438	4.80E-02	**7.96E-04**	**3.25E-03**	5.28E-02	3.38E-03	-	ns
				cg01448863	**4.52E-02**	**1.33E-04**	**1.80E-03**	3.56E-02	1.80E-02	-	ns

SLCO1B1	2	1	50%	cg00995065	5.47E-02	**2.31E-03**	**7.00E-03**	9.38E-02	2.03E-06	-	ns
SLC27A5	13	5	38%	cg19469742	3.92E-02	**2.80E-03**	**8.17E-03**	7.33E-02	3.81E-04	-	ns
				cg18495710	6.32E-02	**3.31E-05**	**1.62E-03**	5.19E-02	1.96E-03	-	ns
				cg16107172	5.07E-02	**8.38E-05**	**1.62E-03**	4.44E-02	4.82E-03	-	ns
				cg07726085	5.10E-02	**1.65E-04**	**1.80E-03**	3.75E-02	1.59E-02	-	ns
				cg16278661	3.88E-02	**5.98E-04**	**2.67E-03**	3.33E-02	1.77E-02	-	ns
SULT2A1	3	1	33%	cg19139729	4.52E-02	**2.05E-04**	**1.80E-03**	3.95E-02	2.17E-02	-	ns
CYP7B1	12	4	33%	cg01510388	5.37E-02	**2.93E-03**	**8.21E-03**	7.16E-02	2.44E-04	-	ns
				cg00054210	6.19E-02	**6.86E-04**	**2.89E-03**	5.84E-02	1.79E-03	-	ns
				cg04418492	1.00E-02	**6.37E-03**	**1.60E-02**	2.17E-02	1.91E-02	-	ns
				cg24990212	2.95E-02	**1.92E-04**	**1.80E-03**	3.18E-02	2.04E-02	-	ns
HSD3B7	10	3	30%	cg07359991	4.66E-02	**1.76E-04**	**1.80E-03**	6.15E-02	2.43E-04	-	ns
				cg10917602	4.41E-02	**1.62E-04**	**1.80E-03**	3.80E-02	2.08E-02	-	ns
				cg09162137	-3.43E-02	**1.69E-03**	**5.55E-03**	-3.20E-02	5.20E-03	-	ns
SCP2	7	2	29%	cg05232215	4.63E-02	**6.61E-04**	**2.83E-03**	6.10E-02	1.06E-04	-	ns
				cg06621784	-4.72E-02	**2.14E-03**	**6.81E-03**	-8.29E-02	2.79E-06	-	ns
CYP27A1	5	1	20%	cg03460682	1.28E-02	**1.69E-03**	**5.55E-03**	3.15E-02	4.23E-03	-	ns
FGF19	17	1	6%	cg24454109	-3.61E-02	**3.38E-03**	**9.34E-03**	-3.06E-02	2.46E-02	-	ns
NR2B1	9	1	11%	cg14651936	-2.38E-02	**2.95E-03**	**8.21E-03**	-3.25E-02	5.67E-05	-	ns
ABCB4	4	1	25%	cg04996195	4.28E-02	**8.05E-03**	**1.96E-02**	6.85E-02	1.94E-05	-	ns
SLCO1A2	4	1	25%	cg16923485	-4.64E-02	**4.59E-04**	**2.39E-03**	-4.34E-02	7.11E-03	-	ns
NR5A2	21	5	24%	cg08158862	3.36E-02	**1.16E-03**	**4.24E-03**	4.73E-02	1.12E-03	-	ns
				cg16993043	2.08E-02	**3.83E-03**	**1.04E-02**	3.88E-02	4.29E-03	-	ns
				cg17804356	1.80E-02	**4.07E-04**	**2.19E-03**	2.68E-02	1.37E-02	-	ns
				cg21851672	-2.89E-02	**3.89E-04**	**2.14E-03**	-3.31E-02	4.85E-04	-	ns
				cg04308769	-5.82E-02	**3.27E-04**	**2.11E-03**	-4.56E-02	1.24E-03	-	ns
ATP8B1	9	2	22%	cg12517027	-8.67E-02	**5.29E-05**	**1.62E-03**	-1.50E-01	1.28E-06	3.55E-02	3.90E-02
				cg21357291	3.93E-02	**3.90E-04**	**2.14E-03**	3.95E-02	1.58E-02	-	ns
FGFR4	12	2	17%	cg00618323	3.01E-02	**1.23E-03**	**4.45E-03**	2.65E-02	2.42E-03	-	ns
				cg04849878	5.45E-02	**3.16E-04**	**2.11E-03**	5.03E-02	3.88E-03	-	ns
ABCC3	8	1	13%	cg27222669	-4.17E-02	**1.07E-04**	**1.62E-03**	-4.86E-02	**1.36E-04**	-	**ns**

Shown are all targets that displayed significantly differentially methylated CpG sites. Listed are the CpG sites that are NAFLD dependently significantly differentially methylated and validated. Targets are ordered according to the percentage of affected CpG sites within the TSS1500 interval.

* percentage of differentially methylated CpG sites in NAFLD within the TSS1500 interval as confirmed in two cohorts.

** The discovery cohort is composed of liver healthy and NAFLD patients. Performance of multiple linear regression analyses investigating the association between methylation state of gene associated individual CpG sites and hepatic disease state. Listed are raw p-values and false discovery (FDR) corrected p-values.

*** The validation cohort is composed of NAFLD patients with different stages of steatosis and fibrosis. Performance of multiple linear regression analyses investigating the association between methylation state of gene associated individuals CpG sites and severity of fibrosis and steatotis, respectively. Listed are raw p-values and FDR corrected p-values.

-, no coefficient given for non significant results; n, number; coef, coefficient; FDR, false discovery rate; sign., significant; total, investigated in total
