# Supplementary material for: A targeted analysis reveals relevant shifts in the methylation and transcription of genes responsible for bile acid homeostasis and drug metabolism in non-alcoholic fatty liver disease
Source: BMC Genomics. 2016 Jun 14;17:462. doi: 10.1186/s12864-016-2814-z (PMC4908840; doi:10.1186/s12864-016-2814-z)
Supplement: Additional file 3: Table S3. — Methylation changes in genes involved in bile acid homeostasis (gene cluster 1) – percent methylation of significantly changed CpG sites in healthy individuals and in patients with different stages of NAFLD. (PDF 204 kb) [file 12864_2016_2814_MOESM3_ESM.pdf]

**A targeted analysis reveals relevant shifts in the methylation and transcription of genes responsible for bile acid homeostasis and drug metabolism in non-alcoholic fatty liver disease**

Supplement

**Authors:** H. B. Schiöth[1], A. Boström[1], S. K. Murphy[2], W. Erhart[3], J. Hampe[4], C. Moylan[5,6] and J. Mwinyi[1]

[1]Department of Neuroscience, Functional Pharmacology, Uppsala University, Uppsala, Sweden

[2]Department of Obstetrics and Gynecology, Duke University Medical Center, Durham, North Carolina, USA

[3]Department of Internal Medicine I, University Hospital Schleswig-Holstein, Kiel, Germany

[4]Medical Department I, University Hospital Dresden, Dresden, Germany

[5]Department of Medicine, Duke University Medical Center, Durham, North Carolina, USA

[6]Department of Medicine, Durham Veterans Affairs Medical Center, Durham, North Carolina, USA

**Additional file 3: Table S3.** Methylation changes in genes involved in bile acid homeostasis (gene cluster 1) – percent methylation of significantly changed CpG sites in healthy individuals and in patients with different stages of NAFLD

**Table S3** Methylation changes in genes involved in bile acid homeostasis – percent methylation of significantly changed CpG sites in healthy individuals and in patients with different stages of NAFLD

| | | | | | Discovery Group (n=74)** | | | | Validation Cohort (n=54)*** | | | |
|---|---|---|---|---|---|---|---|---|---|---|---|---|
| | CpG-sites | | | | Healthy | | NAFLD&NASH | | Mild fibrosis stage | | Advanced fibrosis stage | |
| Gene | Total | Sign. | Sign. (%)* | ID | Mean | SD | Mean | SD | Mean | SD | Mean | SD |
| ***ABCG8*** | 1 | 1 | 100% | cg00760272 | 0.40 | 0.049 | 0.45 | 0.075 | 0.39 | 0.051 | 0.46 | 0.097 |
| ***UGT2B11*** | 1 | 1 | 100% | cg27372994 | 0.26 | 0.031 | 0.28 | 0.044 | 0.26 | 0.037 | 0.30 | 0.056 |
| ***ABCG5*** | 12 | 11 | 92% | cg01186613 | 0.40 | 0.055 | 0.45 | 0.074 | 0.38 | 0.046 | 0.45 | 0.081 |
| | | | | cg00009421 | 0.37 | 0.033 | 0.40 | 0.043 | 0.35 | 0.033 | 0.39 | 0.067 |
| | | | | cg07681696 | 0.43 | 0.039 | 0.47 | 0.055 | 0.42 | 0.044 | 0.47 | 0.075 |
| | | | | cg16451365 | 0.39 | 0.036 | 0.43 | 0.048 | 0.39 | 0.036 | 0.43 | 0.069 |
| | | | | cg11467440 | 0.48 | 0.029 | 0.51 | 0.040 | 0.45 | 0.034 | 0.48 | 0.048 |
| | | | | cg18281102 | 0.38 | 0.038 | 0.42 | 0.049 | 0.37 | 0.037 | 0.41 | 0.075 |
| | | | | cg25781162 | 0.42 | 0.031 | 0.46 | 0.043 | 0.42 | 0.041 | 0.46 | 0.073 |
| | | | | cg00705576 | 0.40 | 0.027 | 0.43 | 0.041 | 0.39 | 0.036 | 0.42 | 0.060 |
| | | | | cg11113753 | 0.37 | 0.029 | 0.40 | 0.042 | 0.35 | 0.028 | 0.38 | 0.057 |
| | | | | cg08453096 | 0.48 | 0.045 | 0.51 | 0.050 | 0.49 | 0.042 | 0.52 | 0.081 |
| | | | | cg13341470 | 0.38 | 0.032 | 0.41 | 0.040 | 0.37 | 0.031 | 0.40 | 0.053 |
| ***HNF1A*** | 8 | 7 | 88% | cg01341572 | 0.47 | 0.031 | 0.50 | 0.048 | 0.39 | 0.042 | 0.44 | 0.072 |
| | | | | cg05934698 | 0.39 | 0.031 | 0.42 | 0.042 | 0.38 | 0.036 | 0.43 | 0.075 |
| | | | | cg25477769 | 0.51 | 0.024 | 0.54 | 0.032 | 0.48 | 0.033 | 0.52 | 0.047 |
| | | | | cg23661013 | 0.41 | 0.038 | 0.45 | 0.053 | 0.39 | 0.042 | 0.43 | 0.064 |
| | | | | cg02153339 | 0.38 | 0.040 | 0.42 | 0.050 | 0.35 | 0.041 | 0.39 | 0.073 |
| | | | | cg14996810 | 0.49 | 0.039 | 0.53 | 0.052 | 0.48 | 0.051 | 0.52 | 0.083 |
| | | | | cg06444781 | 0.46 | 0.026 | 0.49 | 0.039 | 0.45 | 0.046 | 0.49 | 0.070 |
| ***OSTalpha*** | 7 | 6 | 86% | cg17526770 | 0.40 | 0.034 | 0.44 | 0.046 | 0.39 | 0.030 | 0.43 | 0.072 |
| | | | | cg27558485 | 0.37 | 0.031 | 0.40 | 0.042 | 0.36 | 0.037 | 0.40 | 0.072 |
| | | | | cg08877188 | 0.39 | 0.027 | 0.41 | 0.038 | 0.31 | 0.028 | 0.34 | 0.047 |
| | | | | cg21748136 | 0.39 | 0.034 | 0.42 | 0.049 | 0.37 | 0.038 | 0.42 | 0.076 |
| | | | | cg05473677 | 0.35 | 0.029 | 0.37 | 0.041 | 0.34 | 0.025 | 0.37 | 0.060 |
| | | | | cg04478991 | 0.31 | 0.031 | 0.33 | 0.037 | 0.30 | 0.035 | 0.33 | 0.058 |
| ***CYP8B1*** | 6 | 5 | 83% | cg21979032 | 0.35 | 0.035 | 0.38 | 0.049 | 0.33 | 0.035 | 0.38 | 0.062 |
| | | | | cg20365867 | 0.46 | 0.042 | 0.50 | 0.063 | 0.45 | 0.046 | 0.50 | 0.076 |
| | | | | cg26124860 | 0.39 | 0.046 | 0.44 | 0.063 | 0.39 | 0.041 | 0.44 | 0.090 |
| | | | | cg07147573 | 0.38 | 0.041 | 0.41 | 0.053 | 0.36 | 0.039 | 0.40 | 0.079 |
| | | | | cg14404298 | 0.36 | 0.035 | 0.39 | 0.045 | 0.34 | 0.043 | 0.37 | 0.063 |
| ***NR2A1*** | 18 | 15 | 83% | cg27420224 | 0.48 | 0.050 | 0.52 | 0.056 | 0.43 | 0.040 | 0.49 | 0.070 |
| | | | | cg23792485 | 0.52 | 0.045 | 0.56 | 0.054 | 0.48 | 0.050 | 0.53 | 0.072 |
| | | | | cg06640637 | 0.52 | 0.040 | 0.56 | 0.053 | 0.46 | 0.048 | 0.52 | 0.070 |
| | | | | cg08314996 | 0.35 | 0.042 | 0.39 | 0.053 | 0.32 | 0.046 | 0.36 | 0.068 |
| | | | | cg21081369 | 0.37 | 0.029 | 0.40 | 0.036 | 0.34 | 0.031 | 0.38 | 0.056 |
| | | | | cg19717150 | 0.30 | 0.034 | 0.33 | 0.046 | 0.25 | 0.034 | 0.29 | 0.049 |
| | | | | cg07006673 | 0.35 | 0.039 | 0.39 | 0.061 | 0.34 | 0.046 | 0.38 | 0.074 |
| | | | | cg23834593 | 0.34 | 0.036 | 0.37 | 0.046 | 0.32 | 0.036 | 0.35 | 0.058 |

| | | | | | | | | | | | | |
|---|---|---|---|---|---|---|---|---|---|---|---|---|
| | | | | cg03862380 | 0.31 | 0.032 | 0.34 | 0.044 | 0.29 | 0.037 | 0.32 | 0.065 |
| | | | | cg16221969 | 0.44 | 0.029 | 0.46 | 0.038 | 0.42 | 0.040 | 0.45 | 0.056 |
| | | | | cg21559386 | 0.33 | 0.038 | 0.36 | 0.048 | 0.31 | 0.034 | 0.33 | 0.061 |
| | | | | cg06126829 | 0.43 | 0.032 | 0.47 | 0.048 | 0.42 | 0.042 | 0.45 | 0.065 |
| | | | | cg20265805 | 0.34 | 0.030 | 0.36 | 0.036 | 0.32 | 0.033 | 0.34 | 0.048 |
| | | | | cg24084358 | 0.43 | 0.034 | 0.46 | 0.046 | 0.41 | 0.041 | 0.44 | 0.072 |
| ***NR1I3*** | 4 | 3 | 75% | cg06277277 | 0.30 | 0.035 | 0.34 | 0.054 | 0.29 | 0.035 | 0.33 | 0.065 |
| | | | | cg25214346 | 0.31 | 0.029 | 0.34 | 0.042 | 0.31 | 0.028 | 0.34 | 0.064 |
| | | | | cg17028814 | 0.35 | 0.036 | 0.39 | 0.051 | 0.35 | 0.037 | 0.38 | 0.066 |
| ***NR1H4*** | 4 | 3 | 75% | cg01202639 | 0.31 | 0.041 | 0.36 | 0.054 | 0.32 | 0.047 | 0.36 | 0.067 |
| | | | | cg08098450 | 0.46 | 0.052 | 0.51 | 0.074 | 0.49 | 0.054 | 0.56 | 0.082 |
| | | | | cg03771004 | 0.30 | 0.043 | 0.35 | 0.058 | 0.32 | 0.041 | 0.36 | 0.088 |
| ***EPHX1*** | 13 | 9 | 69% | cg26187962 | 0.36 | 0.038 | 0.40 | 0.058 | 0.36 | 0.056 | 0.39 | 0.075 |
| | | | | cg03337430 | 0.52 | 0.043 | 0.57 | 0.072 | 0.54 | 0.053 | 0.59 | 0.079 |
| | | | | cg24928687 | 0.28 | 0.042 | 0.33 | 0.070 | 0.26 | 0.037 | 0.33 | 0.083 |
| | | | | cg17468616 | 0.41 | 0.042 | 0.45 | 0.058 | 0.40 | 0.035 | 0.45 | 0.071 |
| | | | | cg23096144 | 0.25 | 0.028 | 0.28 | 0.048 | 0.23 | 0.024 | 0.27 | 0.052 |
| | | | | cg03459809 | 0.17 | 0.027 | 0.20 | 0.053 | 0.16 | 0.029 | 0.18 | 0.048 |
| | | | | cg05385434 | 0.20 | 0.043 | 0.22 | 0.063 | 0.20 | 0.040 | 0.26 | 0.098 |
| | | | | cg25152404 | 0.28 | 0.031 | 0.31 | 0.044 | 0.26 | 0.030 | 0.30 | 0.073 |
| | | | | cg24868305 | 0.17 | 0.032 | 0.18 | 0.048 | 0.16 | 0.024 | 0.20 | 0.073 |
| ***ABCB11*** | 3 | 2 | 67% | cg20118424 | 0.43 | 0.047 | 0.48 | 0.069 | 0.44 | 0.054 | 0.50 | 0.093 |
| | | | | cg00295325 | 0.30 | 0.036 | 0.33 | 0.055 | 0.32 | 0.036 | 0.36 | 0.071 |
| ***CYP3A4*** | 3 | 2 | 67% | cg23326197 | 0.77 | 0.025 | 0.74 | 0.033 | 0.82 | 0.023 | 0.79 | 0.035 |
| | | | | cg22821554 | 0.85 | 0.017 | 0.84 | 0.024 | 0.89 | 0.021 | 0.87 | 0.029 |
| ***SLCO2B1*** | 9 | 6 | 67% | cg12537437 | 0.39 | 0.040 | 0.44 | 0.076 | 0.37 | 0.047 | 0.45 | 0.082 |
| | | | | cg25367084 | 0.44 | 0.041 | 0.49 | 0.071 | 0.38 | 0.035 | 0.45 | 0.079 |
| | | | | cg23577865 | 0.48 | 0.029 | 0.51 | 0.048 | 0.44 | 0.025 | 0.48 | 0.056 |
| | | | | cg20358275 | 0.44 | 0.032 | 0.46 | 0.050 | 0.39 | 0.038 | 0.43 | 0.055 |
| | | | | cg15751948 | 0.46 | 0.045 | 0.50 | 0.054 | 0.44 | 0.044 | 0.48 | 0.074 |
| | | | | cg18589858 | 0.77 | 0.022 | 0.75 | 0.020 | 0.81 | 0.029 | 0.78 | 0.032 |
| ***ABCC2*** | 5 | 3 | 60% | cg17044311 | 0.38 | 0.050 | 0.42 | 0.070 | 0.37 | 0.050 | 0.44 | 0.093 |
| | | | | cg14947634 | 0.41 | 0.052 | 0.46 | 0.071 | 0.41 | 0.052 | 0.47 | 0.091 |
| | | | | cg09448875 | 0.40 | 0.049 | 0.45 | 0.067 | 0.40 | 0.045 | 0.46 | 0.093 |
| ***NR0B2*** | 5 | 3 | 60% | cg16177693 | 0.29 | 0.033 | 0.32 | 0.049 | 0.28 | 0.030 | 0.32 | 0.065 |
| | | | | cg06650260 | 0.30 | 0.033 | 0.33 | 0.045 | 0.29 | 0.036 | 0.33 | 0.074 |
| | | | | cg16762386 | 0.36 | 0.034 | 0.40 | 0.052 | 0.37 | 0.040 | 0.41 | 0.069 |
| ***NR1I2*** | 5 | 3 | 60% | cg22276685 | 0.37 | 0.038 | 0.40 | 0.052 | 0.35 | 0.037 | 0.40 | 0.077 |
| | | | | cg00836482 | 0.60 | 0.024 | 0.57 | 0.034 | 0.62 | 0.036 | 0.59 | 0.033 |
| | | | | cg25562664 | 0.47 | 0.024 | 0.45 | 0.029 | 0.50 | 0.041 | 0.45 | 0.042 |
| ***BAAT*** | 2 | 1 | 50% | cg05770171 | 0.48 | 0.051 | 0.53 | 0.070 | 0.51 | 0.059 | 0.57 | 0.079 |
| ***SLC10A1*** | 6 | 3 | 50% | cg05633152 | 0.41 | 0.039 | 0.45 | 0.058 | 0.41 | 0.036 | 0.46 | 0.072 |
| | | | | cg21088438 | 0.30 | 0.044 | 0.33 | 0.050 | 0.29 | 0.045 | 0.34 | 0.075 |
| | | | | cg01448863 | 0.25 | 0.035 | 0.27 | 0.045 | 0.25 | 0.033 | 0.28 | 0.069 |
| ***SLCO1B1*** | 2 | 1 | 50% | cg00995065 | 0.36 | 0.050 | 0.41 | 0.073 | 0.38 | 0.052 | 0.46 | 0.074 |
| ***SLC27A5*** | 13 | 5 | 38% | cg19469742 | 0.40 | 0.033 | 0.43 | 0.056 | 0.40 | 0.049 | 0.46 | 0.090 |

| | | | | | | | | | | | | |
|---|---|---|---|---|---|---|---|---|---|---|---|---|
| | | | | cg18495710 | 0.40 | 0.041 | 0.45 | 0.055 | 0.40 | 0.041 | 0.44 | 0.073 |
| | | | | cg16107172 | 0.44 | 0.032 | 0.47 | 0.050 | 0.43 | 0.037 | 0.47 | 0.069 |
| | | | | cg07726085 | 0.40 | 0.033 | 0.43 | 0.053 | 0.37 | 0.039 | 0.40 | 0.068 |
| | | | | cg16278661 | 0.31 | 0.028 | 0.34 | 0.045 | 0.30 | 0.030 | 0.33 | 0.063 |
| ***SULT2A1*** | 3 | 1 | 33% | cg19139729 | 0.32 | 0.032 | 0.36 | 0.046 | 0.31 | 0.040 | 0.34 | 0.076 |
| ***CYP7B1*** | 12 | 4 | 33% | cg01510388 | 0.30 | 0.054 | 0.32 | 0.066 | 0.25 | 0.052 | 0.32 | 0.074 |
| | | | | cg00054210 | 0.26 | 0.054 | 0.29 | 0.067 | 0.20 | 0.049 | 0.25 | 0.074 |
| | | | | cg04418492 | 0.04 | 0.013 | 0.05 | 0.014 | 0.03 | 0.010 | 0.05 | 0.048 |
| | | | | cg24990212 | 0.10 | 0.023 | 0.11 | 0.033 | 0.10 | 0.021 | 0.13 | 0.069 |
| ***HSD3B7*** | 10 | 3 | 30% | cg07359991 | 0.50 | 0.037 | 0.53 | 0.044 | 0.53 | 0.046 | 0.58 | 0.067 |
| | | | | cg10917602 | 0.35 | 0.030 | 0.38 | 0.046 | 0.34 | 0.036 | 0.37 | 0.075 |
| | | | | cg09162137 | 0.61 | 0.031 | 0.59 | 0.039 | 0.69 | 0.040 | 0.65 | 0.036 |
| ***SCP2*** | 7 | 2 | 29% | cg05232215 | 0.14 | 0.037 | 0.16 | 0.055 | 0.09 | 0.021 | 0.14 | 0.074 |
| | | | | cg06621784 | 0.19 | 0.045 | 0.15 | 0.053 | 0.22 | 0.065 | 0.13 | 0.047 |
| ***CYP27A1*** | 5 | 1 | 20% | cg03460682 | 0.09 | 0.011 | 0.10 | 0.019 | 0.09 | 0.016 | 0.12 | 0.054 |
| ***FGF19*** | 17 | 1 | 6% | cg24454109 | 0.67 | 0.034 | 0.64 | 0.046 | 0.72 | 0.040 | 0.69 | 0.050 |
| ***NR2B1*** | 9 | 1 | 11% | cg14651936 | 0.77 | 0.027 | 0.76 | 0.026 | 0.84 | 0.022 | 0.81 | 0.029 |
| ***ABCB4*** | 4 | 1 | 25% | cg04996195 | 0.23 | 0.049 | 0.26 | 0.059 | 0.18 | 0.035 | 0.24 | 0.072 |
| ***SLCO1A2*** | 4 | 1 | 25% | cg16923485 | 0.66 | 0.036 | 0.63 | 0.050 | 0.72 | 0.050 | 0.68 | 0.058 |
| ***NR5A2*** | 21 | 5 | 24% | cg08158862 | 0.19 | 0.026 | 0.22 | 0.040 | 0.20 | 0.044 | 0.23 | 0.058 |
| | | | | cg16993043 | 0.05 | 0.025 | 0.06 | 0.028 | 0.04 | 0.019 | 0.07 | 0.070 |
| | | | | cg17804356 | 0.11 | 0.015 | 0.12 | 0.020 | 0.11 | 0.021 | 0.13 | 0.053 |
| | | | | cg21851672 | 0.55 | 0.028 | 0.54 | 0.025 | 0.58 | 0.031 | 0.55 | 0.029 |
| | | | | cg04308769 | 0.65 | 0.044 | 0.61 | 0.059 | 0.71 | 0.043 | 0.67 | 0.049 |
| ***ATP8B1*** | 9 | 2 | 22% | cg12517027 | 0.60 | 0.063 | 0.54 | 0.093 | 0.66 | 0.075 | 0.53 | 0.133 |
| | | | | cg21357291 | 0.12 | 0.036 | 0.14 | 0.046 | 0.11 | 0.024 | 0.15 | 0.080 |
| ***FGFR4*** | 12 | 2 | 17% | cg00618323 | 0.21 | 0.024 | 0.23 | 0.036 | 0.19 | 0.017 | 0.21 | 0.039 |
| | | | | cg04849878 | 0.25 | 0.038 | 0.29 | 0.061 | 0.25 | 0.038 | 0.29 | 0.076 |
| ***ABCC3*** | 8 | 1 | 13% | cg27222669 | 0.75 | 0.033 | 0.72 | 0.043 | 0.79 | 0.038 | 0.75 | 0.049 |
| ***ABCC4*** | 8 | 1 | 13% | cg24476569 | 0.55 | 0.056 | 0.51 | 0.064 | 0.60 | 0.066 | 0.54 | 0.104 |

Shown are all targets that displayed significantly differentially methylated CpG sites. Listed are the CpG sites that are NAFLD dependently significantly differentially methylated and validated. Targets are ordered according to the percentage of affected CpG sites within the TSS1500 interval.

* Percentage of differentially methylated CpG sites in NAFLD within the TSS1500 interval as confirmed in two cohorts.

** The discovery cohort is composed of liver healthy and NAFLD patients.

*** The validation cohort is composed of NAFLD patients with different stages of steatosis and fibrosis.

Mean, percentage methylation of the CpG site; SD, standard deviation; Total, total number of investigated CpG sites of a gene; n, number; coef, coefficient; FDR, false discovery rate
