# Supplementary material for: A targeted analysis reveals relevant shifts in the methylation and transcription of genes responsible for bile acid homeostasis and drug metabolism in non-alcoholic fatty liver disease
Source: BMC Genomics. 2016 Jun 14;17:462. doi: 10.1186/s12864-016-2814-z (PMC4908840; doi:10.1186/s12864-016-2814-z)
Supplement: Additional file 4: Table S4. — Methylation changes in genes involved in drug transport and metabolisms – percent methylation of significantly changed CpG sites in healthy individuals and in patients with different stages of NAFLD. (PDF 182 kb) [file 12864_2016_2814_MOESM4_ESM.pdf]

A targeted analysis reveals relevant shifts in the methylation and transcription of genes responsible for bile acid homeostasis and drug metabolism in non-alcoholic fatty liver disease

Supplement

Authors: H. B. Schiöth[1], A. Boström[1], S. K. Murphy[2], W. Erhart[3], J. Hampe[4], C. Moylan[5,6] and J. Mwinyi[1]

[1]Department of Neuroscience, Functional Pharmacology, Uppsala University, Uppsala, Sweden

[2]Department of Obstetrics and Gynecology, Duke University Medical Center, Durham, North Carolina, USA

[3]Department of Internal Medicine I, University Hospital Schleswig-Holstein, Kiel, Germany

[4]Medical Department I, University Hospital Dresden, Dresden, Germany

[5]Department of Medicine, Duke University Medical Center, Durham, North Carolina, USA

[6]Department of Medicine, Durham Veterans Affairs Medical Center, Durham, North Carolina, USA

Additional file 4: Table S4. Methylation changes in genes involved in drug transport and metabolisms – percent methylation of significantly changed CpG sites in healthy individuals and in patients with different stages of NAFLD

Table S4 Methylation changes in genes involved in drug transport and metabolisms – percent methylation of significantly changed CpG sites in healthy individuals and in patients with different stages of NAFLD

					Discovery Group (n=74)**				Validation Cohort (n=54)***			
	CpG-sites				Healthy		NAFLD&NASH		Mild fibrosis stage		Advanced fibrosis stage	
Gene	Total	Sign.	Sign. (%)*	ID	Mean	SD	Mean	SD	Mean	SD	Mean	SD
CYP2C9	2	2	100%	cg24140586	0.48	0.050	0.52	0.078	0.49	0.061	0.56	0.091
				cg09550024	0.36	0.043	0.39	0.065	0.35	0.042	0.39	0.069
HNF1A	8	8	100%	cg01341572	0.47	0.031	0.50	0.048	0.39	0.042	0.44	0.072
				cg05934698	0.39	0.031	0.42	0.042	0.38	0.036	0.43	0.075
				cg25477769	0.51	0.024	0.54	0.032	0.48	0.033	0.52	0.047
				cg23661013	0.41	0.038	0.45	0.053	0.39	0.042	0.43	0.064
				cg02153339	0.38	0.040	0.42	0.050	0.35	0.041	0.39	0.073
				cg01394199	0.51	0.025	0.53	0.033	0.45	0.033	0.49	0.051
				cg14996810	0.49	0.039	0.53	0.052	0.48	0.051	0.52	0.083
				cg06444781	0.46	0.026	0.49	0.039	0.45	0.046	0.49	0.070
SLCO1B1	1	1	100%	cg00995065	0.36	0.050	0.41	0.073	0.38	0.052	0.46	0.074
SLC22A1	3	3	100%	cg13434757	0.47	0.067	0.52	0.079	0.49	0.069	0.57	0.100
				cg24864413	0.39	0.034	0.42	0.048	0.38	0.036	0.42	0.067
				cg13466809	0.35	0.030	0.38	0.039	0.32	0.030	0.35	0.052
SLC22A7	2	2	100%	cg16465769	0.42	0.027	0.45	0.045	0.40	0.034	0.43	0.065
				cg03990905	0.24	0.028	0.27	0.044	0.24	0.030	0.27	0.057
UGT1A1	2	2	100%	cg19146119	0.64	0.061	0.70	0.055	0.70	0.087	0.77	0.062
				cg07823755	0.83	0.018	0.83	0.018	0.91	0.016	0.89	0.018
CYP2E1	7	6	86%	cg21024264	0.38	0.044	0.42	0.064	0.36	0.046	0.42	0.089
				cg09208540	0.40	0.049	0.44	0.074	0.41	0.059	0.47	0.095
				cg07381788	0.38	0.047	0.43	0.063	0.37	0.049	0.42	0.085
				cg01465364	0.38	0.041	0.42	0.050	0.39	0.044	0.43	0.074
				cg00436603	0.35	0.039	0.38	0.051	0.35	0.033	0.39	0.064
				cg14250048	0.36	0.038	0.39	0.057	0.36	0.050	0.40	0.073
NR2A1	18	15	83%	cg27420224	0.48	0.050	0.52	0.056	0.43	0.040	0.49	0.070
				cg23792485	0.52	0.045	0.56	0.054	0.48	0.050	0.53	0.072
				cg06640637	0.52	0.040	0.56	0.053	0.46	0.048	0.52	0.070
				cg20848979	0.55	0.034	0.58	0.047	0.50	0.043	0.54	0.057
				cg08314996	0.35	0.042	0.39	0.053	0.32	0.046	0.36	0.068
				cg21081369	0.37	0.029	0.40	0.036	0.34	0.031	0.38	0.056
				cg19717150	0.30	0.034	0.33	0.046	0.25	0.034	0.29	0.049
				cg07006673	0.35	0.039	0.39	0.061	0.34	0.046	0.38	0.074
				cg23834593	0.34	0.036	0.37	0.046	0.32	0.036	0.35	0.058
				cg03862380	0.31	0.032	0.34	0.044	0.29	0.037	0.32	0.065
				cg16221969	0.44	0.029	0.46	0.038	0.42	0.040	0.45	0.056
				cg21559386	0.33	0.038	0.36	0.048	0.31	0.034	0.33	0.061
				cg06126829	0.43	0.032	0.47	0.048	0.42	0.042	0.45	0.065

				cg20265805	0.34	0.030	0.36	0.036	0.32	0.033	0.34	0.048
				cg24084358	0.43	0.034	0.46	0.046	0.41	0.041	0.44	0.072
UGT1A4	5	4	80%	cg04437648	0.47	0.053	0.53	0.074	0.40	0.046	0.48	0.089
				cg05313279	0.45	0.054	0.50	0.063	0.47	0.060	0.54	0.099
				cg02234120	0.82	0.029	0.80	0.035	0.87	0.023	0.84	0.040
				cg05141602	0.77	0.036	0.76	0.028	0.83	0.032	0.81	0.038
NR1I3	4	3	75%	cg06277277	0.30	0.035	0.34	0.054	0.29	0.035	0.33	0.065
				cg25214346	0.31	0.029	0.34	0.042	0.31	0.028	0.34	0.064
				cg17028814	0.35	0.036	0.39	0.051	0.35	0.037	0.38	0.066
CYP1A2	3	2	67%	cg11473616	0.42	0.051	0.47	0.070	0.43	0.055	0.49	0.080
				cg04968473	0.52	0.070	0.60	0.070	0.59	0.086	0.68	0.086
CYP2D6	3	2	67%	cg09322432	0.46	0.045	0.51	0.061	0.47	0.061	0.54	0.089
				cg04692870	0.37	0.048	0.40	0.030	0.37	0.052	0.42	0.070
CYP3A4	3	2	67%	cg23326197	0.77	0.025	0.74	0.033	0.82	0.023	0.79	0.035
				cg22821554	0.85	0.017	0.84	0.024	0.89	0.021	0.87	0.029
SLCO2B1	9	6	67%	cg12537437	0.39	0.040	0.44	0.076	0.37	0.047	0.45	0.082
				cg25367084	0.44	0.041	0.49	0.071	0.38	0.035	0.45	0.079
				cg23577865	0.48	0.029	0.51	0.048	0.44	0.025	0.48	0.056
				cg20358275	0.44	0.032	0.46	0.050	0.39	0.038	0.43	0.055
				cg15751948	0.46	0.045	0.50	0.054	0.44	0.044	0.48	0.074
				cg18589858	0.77	0.022	0.75	0.020	0.81	0.029	0.78	0.032
ABCC2	5	3	60%	cg17044311	0.38	0.050	0.42	0.070	0.37	0.050	0.44	0.093
				cg14947634	0.41	0.052	0.46	0.071	0.41	0.052	0.47	0.091
				cg09448875	0.40	0.049	0.45	0.067	0.40	0.045	0.46	0.093
NR1I2	5	3	60%	cg22276685	0.37	0.038	0.40	0.052	0.35	0.037	0.40	0.077
				cg00836482	0.60	0.024	0.57	0.034	0.62	0.036	0.59	0.033
				cg25562664	0.47	0.024	0.45	0.029	0.50	0.041	0.45	0.042
CYP2C19	6	3	50%	cg00051662	0.53	0.100	0.61	0.114	0.59	0.120	0.73	0.120
				cg16227251	0.38	0.052	0.43	0.061	0.42	0.076	0.48	0.096
				cg04189838	0.65	0.107	0.74	0.100	0.67	0.134	0.82	0.107
NAT2	2	1	50%	cg12639234	0.43	0.036	0.47	0.062	0.44	0.057	0.50	0.080
UGT2B7	2	1	50%	cg25583503	0.31	0.043	0.35	0.056	0.34	0.055	0.39	0.083
NR3A1	47	23	49%	cg07671949	0.62	0.069	0.59	0.080	0.73	0.052	0.62	0.090
				cg20627916	0.58	0.074	0.54	0.083	0.71	0.061	0.60	0.098
				cg19411146	0.52	0.070	0.48	0.085	0.58	0.070	0.48	0.093
				cg21608605	0.67	0.050	0.62	0.074	0.75	0.042	0.67	0.062
				cg15626350	0.76	0.035	0.74	0.032	0.83	0.030	0.82	0.038
				cg24900983	0.53	0.043	0.50	0.053	0.61	0.046	0.55	0.070
				cg23165623	0.48	0.068	0.46	0.078	0.59	0.064	0.50	0.087
				cg15980539	0.57	0.023	0.54	0.035	0.59	0.034	0.55	0.034
				cg05171584	0.47	0.043	0.44	0.051	0.55	0.048	0.47	0.068
				cg01777019	0.55	0.055	0.52	0.073	0.63	0.057	0.53	0.092
				cg00655307	0.52	0.058	0.48	0.074	0.59	0.062	0.50	0.083
				cg21950534	0.47	0.061	0.45	0.068	0.57	0.065	0.47	0.082
				cg00920970	0.63	0.038	0.59	0.044	0.64	0.050	0.60	0.043
				cg19449067	0.28	0.054	0.31	0.064	0.33	0.056	0.43	0.106

				cg07746998	0.34	0.037	0.38	0.060	0.34	0.041	0.40	0.063
				cg08884395	0.42	0.048	0.47	0.057	0.48	0.048	0.53	0.077
				cg04211581	0.60	0.044	0.56	0.065	0.69	0.036	0.62	0.063
				cg20253551	0.55	0.038	0.51	0.047	0.59	0.051	0.53	0.057
				cg18007957	0.63	0.038	0.58	0.063	0.72	0.041	0.65	0.057
				cg08907436	0.89	0.016	0.88	0.018	0.93	0.014	0.92	0.016
				cg22157087	0.71	0.046	0.68	0.056	0.78	0.040	0.72	0.046
				cg02720618	0.70	0.033	0.67	0.047	0.81	0.039	0.77	0.044
				cg08161546	0.72	0.039	0.69	0.051	0.80	0.033	0.76	0.042
SULT1A1	7	3	43%	cg01378222	0.45	0.053	0.50	0.066	0.45	0.069	0.55	0.130
				cg09685060	0.23	0.039	0.25	0.060	0.18	0.027	0.23	0.056
				cg08008286	0.47	0.045	0.51	0.043	0.49	0.067	0.53	0.087
SLC47A1	15	6	40%	cg08895056	0.38	0.040	0.41	0.041	0.36	0.039	0.43	0.080
				cg10718608	0.40	0.035	0.42	0.033	0.38	0.039	0.43	0.070
				cg01530032	0.32	0.050	0.35	0.060	0.29	0.054	0.38	0.099
				cg12133118	0.28	0.030	0.30	0.039	0.25	0.034	0.29	0.055
				cg24151087	0.13	0.021	0.14	0.022	0.11	0.018	0.13	0.031
				cg15014549	0.07	0.008	0.07	0.008	0.06	0.009	0.07	0.042
CYP2B6	8	3	38%	cg19756068	0.38	0.038	0.42	0.053	0.36	0.035	0.40	0.066
				cg10322876	0.48	0.036	0.51	0.061	0.47	0.052	0.51	0.068
				cg08852641	0.64	0.044	0.61	0.045	0.73	0.034	0.68	0.059
CYP1A1	17	6	35%	cg12101586	0.16	0.035	0.20	0.065	0.11	0.039	0.21	0.114
				cg13570656	0.15	0.045	0.21	0.076	0.12	0.064	0.25	0.155
				cg00213123	0.18	0.040	0.21	0.065	0.15	0.068	0.24	0.095
				cg26516004	0.47	0.047	0.52	0.070	0.42	0.069	0.55	0.112
				cg17852385	0.21	0.044	0.26	0.066	0.21	0.071	0.34	0.151
				cg11924019	0.31	0.035	0.34	0.052	0.30	0.050	0.39	0.105
SLC22A2	10	3	30%	cg04294894	0.83	0.019	0.81	0.018	0.88	0.019	0.85	0.037
				cg07026448	0.65	0.036	0.62	0.037	0.70	0.034	0.66	0.043
				cg19627213	0.67	0.036	0.64	0.043	0.72	0.045	0.67	0.055
SLCO1A2	4	1	25%	cg16923485	0.66	0.036	0.63	0.050	0.72	0.050	0.68	0.058
SLC22A6	4	1	25%	cg04458776	0.73	0.037	0.71	0.033	0.79	0.037	0.75	0.062
NR3C1	15	2	13%	cg07733851	0.11	0.019	0.13	0.033	0.11	0.022	0.13	0.043
				cg08845721	0.26	0.045	0.30	0.063	0.27	0.045	0.32	0.075
SLC22A8	8	1	13%	cg15572436	0.60	0.065	0.55	0.071	0.62	0.064	0.53	0.097
NR2B1	9	1	11%	cg14651936	0.77	0.027	0.76	0.026	0.84	0.022	0.81	0.029
GSTP1	10	1	10%	cg06928838	0.46	0.058	0.43	0.066	0.49	0.070	0.42	0.096

Shown are all targets that displayed significantly differentially methylated CpG sites. Listed are the CpG sites that are NAFLD dependently significantly differentially methylated and validated. Targets are ordered according to the percentage of affected CpG sites within the TSS1500 interval.

* Percentage of differentially methylated CpG sites in NAFLD within the TSS1500 interval as confirmed in two cohorts.

** The discovery cohort is composed of liver healthy and NAFLD patients.

*** The validation cohort is composed of NAFLD patients with different stages of steatosis and fibrosis.

Mean, percentage methylation of the CpG site; SD, standard deviation; Total, total number of investigated CpG sites of a gene; n, number; coef, coefficient; FDR, false discovery rate; sign., significant; total, investigated in total
