# Supplementary material for: A targeted analysis reveals relevant shifts in the methylation and transcription of genes responsible for bile acid homeostasis and drug metabolism in non-alcoholic fatty liver disease
Source: BMC Genomics. 2016 Jun 14;17:462. doi: 10.1186/s12864-016-2814-z (PMC4908840; doi:10.1186/s12864-016-2814-z)
Supplement: Additional file 5: Table S5. — NAFLD dependent associations between the methylation of CpG sites belonging to genes listed in Table 5 and 6 and transcripts of a maximum of 10 neighboring genes. (PDF 226 kb) [file 12864_2016_2814_MOESM5_ESM.pdf]

A targeted analysis reveals relevant shifts in the methylation and transcription of genes responsible for bile acid homeostasis and drug metabolism in non-alcoholic fatty liver disease

Supplement

Authors: H. B. Schiöth[1], A. Boström[1], S. K. Murphy[2], W. Erhart[3], J. Hampe[4], C. Moylan[5,6] and J. Mwinyi[1]

[1]Department of Neuroscience, Functional Pharmacology, Uppsala University, Uppsala, Sweden

[2]Department of Obstetrics and Gynecology, Duke University Medical Center, Durham, North Carolina, USA

[3]Department of Internal Medicine I, University Hospital Schleswig-Holstein, Kiel, Germany

[4]Medical Department I, University Hospital Dresden, Dresden, Germany

[5]Department of Medicine, Duke University Medical Center, Durham, North Carolina, USA

[6]Department of Medicine, Durham Veterans Affairs Medical Center, Durham, North Carolina, USA

Additional file 5: Table S5. NAFLD dependent associations between the methylation of CpG sites belonging to genes listed in table 5 and 6 and transcripts of a maximum of 10 neighboring genes

Table S5 NAFLD dependent associations between the methylation of CpG sites belonging to genes listed in table 5 and 6 and transcripts of a maximum of 10 neighboring genes

					Robust Linear Regression Model** (n=50)				Pearson Correlation***			
									NAFLD & NASH (n=20)		Controls (n=30)	
**No.***	**Gene**	**Neighboring Genes**	**Up/Downstream**	**Affymetrix ID**	***p* (Expr.)**	**Coef. (Expr.)**	***p* (Expr.*Fib)**	**Coef. (Expr.*Fib)**	***p* (Expr.)**	**Coef. (Expr.)**	***p* (Expr.)**	**Coef. (Expr.)**
**1.1**	CYP1A2	**CYP1A1**	Downstream	7990391	**1.20E-03**	-0.18	*ns*	-	*ns*	-	*ns*	-
**1.2**	CYP1A2	**EDC3**	Downstream	7990379	*ns*	-	*ns*	-	*ns*	-	**3.94E-02**	0.38
**1.3**	CYP1A2	**CLK3**	Downstream	7984846	*ns*	-	*ns*	-	*ns*	-	*ns*	-
**1.4**	CYP1A2	**ARID3B**	Downstream	7902592	*ns*	-	*ns*	-	*ns*	-	*ns*	-
**1.5**	CYP1A2	**UBL7**	Downstream	7990361	**1.16E-02**	-0.67	*ns*	-	*ns*	-	*ns*	-
**1.6**	CYP1A2	**CSK**	Upstream	7984871	**1.96E-03**	-0.83	*ns*	-	*ns*	-	*ns*	-
**1.7**	CYP1A2	**LMAN1L**	Upstream	7984892	*ns*	-	*ns*	-	*ns*	-	*ns*	-
**1.8**	CYP1A2	**CPLX3**	Upstream	7984908	*ns*	-	*ns*	-	*ns*	-	*ns*	-
**1.9**	CYP1A2	**ULK3**	Upstream	7990400	*ns*	-	*ns*	-	*ns*	-	*ns*	-
**1.10**	CYP1A2	**SCAMP2**	Upstream	7990417	**4.78E-02**	-0.48	*ns*	-	*ns*	-	*ns*	-
**2.1**	CYP27A1	**TTLL4**	Downstream	8048411	*ns*	-	*ns*	-	*ns*	-	*ns*	-
**2.2**	CYP27A1	**STK36**	Downstream	8048381	*ns*	-	*ns*	-	*ns*	-	*ns*	-
**2.3**	CYP27A1	**BCS1L**	Downstream	8048370	*ns*	-	*ns*	-	*ns*	-	**7.04E-03**	-0.48
**2.4**	CYP27A1	**PLCD4**	Downstream	8048350	*ns*	-	*ns*	-	*ns*	-	*ns*	-
**2.5**	CYP27A1	**PRKAG3**	Upstream	8058997	*ns*	-	*ns*	-	*ns*	-	*ns*	-
**2.6**	CYP27A1	**WNT6**	Upstream	8048445	*ns*	-	*ns*	-	*ns*	-	*ns*	-
**2.7**	CYP27A1	**CDK5R2**	Upstream	8048460	**2.03E-02**	-0.41	*ns*	-	*ns*	-	*ns*	-
**2.8**	CYP27A1	**WNT10A**	Upstream	8048452	*ns*	-	*ns*	-	*ns*	-	*ns*	-
**3.1**	CYP2B6	**CYP2A7**	Downstream	8036981	*ns*	-	*ns*	-	*ns*	-	*ns*	-
**3.2**	CYP2B6	**CYP2A6**	Downstream	8028973	*ns*	-	*ns*	-	*ns*	-	*ns*	-
**3.3**	CYP2B6	**EGLN2**	Downstream	8028940	*ns*	-	*ns*	-	*ns*	-	*ns*	-
**3.4**	CYP2B6	**RAB4B**	Downstream	8028930	*ns*	-	*ns*	-	**2.69E-02**	-0.49	*ns*	-
**3.5**	CYP2B6	**C19orf54**	Downstream	8036956	*ns*	-	*ns*	-	*ns*	-	*ns*	-
**3.6**	CYP2B6	**CYP2A13**	Upstream	8028973	*ns*	-	*ns*	-	*ns*	-	*ns*	-
**3.7**	CYP2B6	**CYP2F1**	Upstream	8028984	*ns*	-	**5.92E-03**	-2.45	*ns*	-	*ns*	-
**3.8**	CYP2B6	**CYP2S1**	Upstream	8028991	*ns*	-	*ns*	-	*ns*	-	*ns*	-
**3.9**	CYP2B6	**AXL**	Upstream	8029006	*ns*	-	*ns*	-	*ns*	-	*ns*	-
**3.10**	CYP2B6	**HNRNPUL1**	Upstream	8029029	*ns*	-	*ns*	-	*ns*	-	*ns*	-
**4.1**	CYP2C19	**CYP2C18**	Downstream	7929466	*ns*	-	*ns*	-	*ns*	-	*ns*	-
**4.2**	CYP2C19	**HELLS**	Downstream	7929438	*ns*	-	*ns*	-	*ns*	-	*ns*	-
**4.3**	CYP2C19	**TBC1D12**	Downstream	7929424	*ns*	-	*ns*	-	*ns*	-	*ns*	-
**4.4**	CYP2C19	**NOC3L**	Downstream	7935146	*ns*	-	*ns*	-	*ns*	-	*ns*	-
**4.5**	CYP2C19	**PLCE1**	Downstream	7929388	*ns*	-	*ns*	-	*ns*	-	*ns*	-
**4.6**	CYP2C19	**CYP2C8**	Upstream	7935169	*ns*	-	*ns*	-	*ns*	-	*ns*	-
**4.7**	CYP2C19	**CYP2C9**	Upstream	7929487	*ns*	-	*ns*	-	*ns*	-	*ns*	-
**4.8**	CYP2C19	**PDLIM1**	Upstream	7935180	*ns*	-	*ns*	-	*ns*	-	*ns*	-
**4.9**	CYP2C19	**SORBS1**	Upstream	7935188	*ns*	-	*ns*	-	**1.74E-03**	0.65	*ns*	-
**4.10**	CYP2C19	**ENTPD1**	Upstream	7929511	*ns*	-	*ns*	-	**2.96E-02**	0.49	*ns*	-
**5.1**	EPHX1	**LBR**	Downstream	7910096	*ns*	-	**2.34E-02**	-2.39	*ns*	-	*ns*	-
**5.2**	EPHX1	**DNAH14**	Downstream	7910030	*ns*	-	**8.15E-03**	13.12	*ns*	-	*ns*	-
**5.3**	EPHX1	**WDR26**	Downstream	7924582	**3.46E-02**	0.42	*ns*	-	*ns*	-	**2.61E-02**	0.41
**5.4**	EPHX1	**PYCR2**	Upstream	7924669	*ns*	-	*ns*	-	*ns*	-	*ns*	-
**5.5**	EPHX1	**TMEM63A**	Upstream	7924636	*ns*	-	*ns*	-	*ns*	-	*ns*	-
**5.6**	EPHX1	**LEFTY1**	Upstream	7924663	**2.83E-02**	-0.35	*ns*	-	*ns*	-	*ns*	-
**5.7**	EPHX1	**LEFTY2**	Upstream	7924682	*ns*	-	*ns*	-	*ns*	-	*ns*	-
**6.1**	FGFR4	**ZNF346**	Downstream	8110253	*ns*	-	*ns*	-	*ns*	-	*ns*	-
**6.2**	FGFR4	**UIMC1**	Downstream	8115978	**3.96E-03**	0.57	*ns*	-	*ns*	-	*ns*	-
**6.3**	FGFR4	**UNC5A**	Downstream	8110237	*ns*	-	*ns*	-	*ns*	-	*ns*	-
**6.4**	FGFR4	**TSPAN17**	Downstream	8110224	*ns*	-	*ns*	-	*ns*	-	*ns*	-
**6.5**	FGFR4	**EIF4E1B**	Downstream	8110218	*ns*	-	*ns*	-	*ns*	-	*ns*	-
**6.6**	FGFR4	**NSD1**	Upstream	8110289	*ns*	-	*ns*	-	*ns*	-	*ns*	-
**6.7**	FGFR4	**RAB24**	Upstream	8115997	*ns*	-	*ns*	-	**2.29E-03**	0.64	*ns*	-

**6.8**	FGFR4	**PRELID1**	Upstream	8110318	**6.97E-03**	-0.28	*ns*	-	*ns*	-	*ns*	-
**6.9**	FGFR4	**MXD3**	Upstream	8116012	*ns*	-	*ns*	-	*ns*	-	*ns*	-
**6.10**	FGFR4	**LMAN2**	Upstream	8116020	*ns*	-	*ns*	-	**2.50E-02**	0.50	*ns*	-
**7.1**	GSTP1	**CABP2**	Downstream	7949843	*ns*	-	*ns*	-	*ns*	-	*ns*	-
**7.2**	GSTP1	**CDK2AP2**	Downstream	7949836	*ns*	-	*ns*	-	*ns*	-	*ns*	-
**7.3**	GSTP1	**PITPNM1**	Downstream	7949808	*ns*	-	*ns*	-	*ns*	-	*ns*	-
**7.4**	GSTP1	**AIP**	Downstream	7949863	*ns*	-	*ns*	-	*ns*	-	*ns*	-
**7.5**	GSTP1	**NDUFV1**	Upstream	7941946	*ns*	-	*ns*	-	*ns*	-	*ns*	-
**7.6**	GSTP1	**NUDT8**	Upstream	7949857	**1.20E-04**	0.42	*ns*	-	*ns*	-	**2.60E-02**	0.41
**7.7**	GSTP1	**TBX10**	Upstream	7949863	*ns*	-	*ns*	-	*ns*	-	*ns*	-
**9.1**	SLC10A1	**SLC39A9**	Downstream	7975344	*ns*	-	*ns*	-	*ns*	-	*ns*	-
**9.2**	SLC10A1	**ERH**	Downstream	7979864	*ns*	-	*ns*	-	*ns*	-	*ns*	-
**9.3**	SLC10A1	**SMOC1**	Upstream	7975390	*ns*	-	*ns*	-	*ns*	-	*ns*	-
**9.4**	SLC10A1	**SLC8A3**	Upstream	7979888	*ns*	-	*ns*	-	*ns*	-	*ns*	-
**9.5**	SLC10A1	**COX16**	Upstream	7979906	*ns*	-	*ns*	-	*ns*	-	*ns*	-
**9.6**	SLC10A1	**ADAM21P1**	Upstream	7975406	*ns*	-	*ns*	-	*ns*	-	*ns*	-
**9.7**	SLC10A1	**SYNJ2BP**	Upstream	7979916	*ns*	-	*ns*	-	*ns*	-	*ns*	-
**10.1**	SLC27A5	**ZNF446**	Downstream	8031962	*ns*	-	*ns*	-	*ns*	-	*ns*	-
**10.2**	SLC27A5	**ZNF324**	Downstream	8031956	*ns*	-	*ns*	-	*ns*	-	*ns*	-
**10.3**	SLC27A5	**ZNF324B**	Downstream	8031949	**1.54E-02**	-0.38	*ns*	-	*ns*	-	*ns*	-
**10.4**	SLC27A5	**ZNF132**	Downstream	8039771	*ns*	-	*ns*	-	*ns*	-	*ns*	-
**10.5**	SLC27A5	**ZNF584**	Downstream	8031939	*ns*	-	*ns*	-	*ns*	-	*ns*	-
**10.6**	SLC27A5	**ZBTB45**	Upstream	8039791	*ns*	-	*ns*	-	*ns*	-	*ns*	-
**10.7**	SLC27A5	**TRIM28**	Upstream	8031913	*ns*	-	*ns*	-	*ns*	-	*ns*	-
**10.8**	SLC27A5	**CHMP2A**	Upstream	8039796	*ns*	-	*ns*	-	*ns*	-	*ns*	-
**10.9**	SLC27A5	**UBE2M**	Upstream	8001111	*ns*	-	**1.21E-04**	-4.44	*ns*	-	*ns*	-
**11.1**	SLC47A1	**RNF112**	Downstream	8005586	*ns*	-	*ns*	-	*ns*	-	*ns*	-
**11.2**	SLC47A1	**B9D1**	Downstream	8013331	*ns*	-	*ns*	-	*ns*	-	*ns*	-
**11.3**	SLC47A1	**MFAP4**	Downstream	8013341	*ns*	-	*ns*	-	*ns*	-	*ns*	-
**11.4**	SLC47A1	**MAPK7**	Downstream	8005576	*ns*	-	*ns*	-	*ns*	-	**8.21E-03**	-0.47
**11.5**	SLC47A1	**MFAP4**	Downstream	8013341	*ns*	-	*ns*	-	*ns*	-	*ns*	-
**11.6**	SLC47A1	**ALDH3A2**	Upstream	8005638	**5.81E-04**	-0.74	*ns*	-	*ns*	-	**2.43E-03**	-0.53
**11.7**	SLC47A1	**SLC47A2**	Upstream	8013364	*ns*	-	*ns*	-	*ns*	-	*ns*	-
**11.8**	SLC47A1	**ALDH3A1**	Upstream	8013384	*ns*	-	*ns*	-	*ns*	-	*ns*	-
**11.9**	SLC47A1	**ULK2**	Upstream	8013399	*ns*	-	*ns*	-	*ns*	-	*ns*	-
**11.10**	SLC47A1	**AKAP10**	Upstream	8013431	**1.05E-04**	1.08	*ns*	-	*ns*	-	*ns*	-
**12.1**	SLC51A	**TFRC**	Downstream	8093053	*ns*	-	*ns*	-	**3.68E-02**	0.47	**1.04E-02**	0.46
**12.2**	SLC51A	**TNK2**	Downstream	8093013	*ns*	-	*ns*	-	*ns*	-	*ns*	-
**12.3**	SLC51A	**MUC4**	Downstream	8092978	*ns*	-	*ns*	-	*ns*	-	*ns*	-
**12.4**	SLC51A	**MUC20**	Downstream	8084895	*ns*	-	*ns*	-	*ns*	-	*ns*	-
**12.5**	SLC51A	**PCYT1A**	Upstream	8093086	*ns*	-	*ns*	-	*ns*	-	*ns*	-
**12.6**	SLC51A	**TCTEX1D2**	Upstream	8093096	*ns*	-	*ns*	-	*ns*	-	*ns*	-
**12.7**	SLC51A	**TM4SF19**	Upstream	8093104	*ns*	-	*ns*	-	*ns*	-	*ns*	-
**12.8**	SLC51A	**RNF168**	Upstream	8093130	*ns*	-	*ns*	-	*ns*	-	*ns*	-
**13.1**	SLCO2B1	**NEU3**	Downstream	7942562	*ns*	-	*ns*	-	*ns*	-	*ns*	-
**13.2**	SLCO2B1	**SPCS2**	Downstream	7914180	*ns*	-	*ns*	-	*ns*	-	*ns*	-
**13.3**	SLCO2B1	**XRRA1**	Downstream	7942544	*ns*	-	*ns*	-	*ns*	-	*ns*	-
**13.4**	SLCO2B1	**RNF169**	Downstream	7942544	*ns*	-	*ns*	-	*ns*	-	*ns*	-
**13.5**	SLCO2B1	**CHRDL2**	Downstream	7950425	*ns*	-	**3.35E-06**	7.11	*ns*	-	*ns*	-
**13.6**	SLCO2B1	**RPS3**	Upstream	7896740	*ns*	-	*ns*	-	*ns*	-	*ns*	-
**13.7**	SLCO2B1	**KLHL35**	Upstream	7950492	*ns*	-	*ns*	-	*ns*	-	*ns*	-
**13.8**	SLCO2B1	**GDPD5**	Upstream	7950501	*ns*	-	**6.13E-06**	-3.79	*ns*	-	*ns*	-
**13.9**	SLCO2B1	**SERPINH1**	Upstream	7942596	*ns*	-	*ns*	-	*ns*	-	*ns*	-
**14.1**	SULT1A1	**SULT1A2**	Downstream	8000582	**1.80E-06**	-0.30	**3.67E-04**	-1.39	*ns*	-	**7.02E-04**	-0.58
**14.2**	SULT1A1	**NUPR1**	Downstream	8000574	*ns*	-	*ns*	-	*ns*	-	**3.24E-03**	-0.52
**14.3**	SULT1A1	**IL27**	Downstream	8000567	*ns*	-	*ns*	-	*ns*	-	**4.98E-02**	-0.36
**14.4**	SULT1A1	**CLN3**	Downstream	8000543	**1.46E-02**	-0.83	*ns*	-	*ns*	-	**3.56E-04**	-0.61
**14.5**	SULT1A1	**EIF3C**	Upstream	7994415	*ns*	-	*ns*	-	*ns*	-	*ns*	-
**14.6**	SULT1A1	**ATXN2L**	Upstream	7994386	*ns*	-	*ns*	-	*ns*	-	*ns*	-
**14.7**	SULT1A1	**TUFM**	Upstream	8000603	*ns*	-	*ns*	-	*ns*	-	*ns*	-

Shown are genes that showed NAFLD dependent methylation transcription associations as listed in table 5 and 6 (alphabetical order). The expression of up to five upstream and 5 downstream located genes (if available and measured by the affymetrix array) was lineary regressed to the average methylation changes of the CpG sites associated to the key genes listed in column 2.

* The number allocates a running number to the key gene investigated and listed in table 5 followed by a running number for the neigbouring gene investigated here with regard to associations between methylation and transcriptional expression.

**Regression analyses studying the association between average methylation in the TSS1500 interval of the gene listed in column 2 and transcription of the neigbouring genes listed in column 3 in the EC cohort. Analyses are taking the strength of fibrosis into account either as independent covariate (column 6 and 7) or as interaction term that combines fibrosis and expression (column 8 and 9).

***Pearson`s correlation analyses correlating average methylation state in the TSS1500 interval and strength of transcription in NAFLD and non-NAFLD patients of the EC cohort

-, no coefficient given for non significant results;No, number; ns, not significant; coef, coefficient; ID, identification number
